# Multi-Level Computational Screening of *in Silico* Designed MOFs for Efficient SO_2_ Capture

**DOI:** 10.1021/acs.jpcc.2c00227

**Published:** 2022-06-03

**Authors:** Hakan Demir, Seda Keskin

**Affiliations:** Department of Chemical and Biological Engineering, Koc University, 34450 Istanbul, Turkey

## Abstract

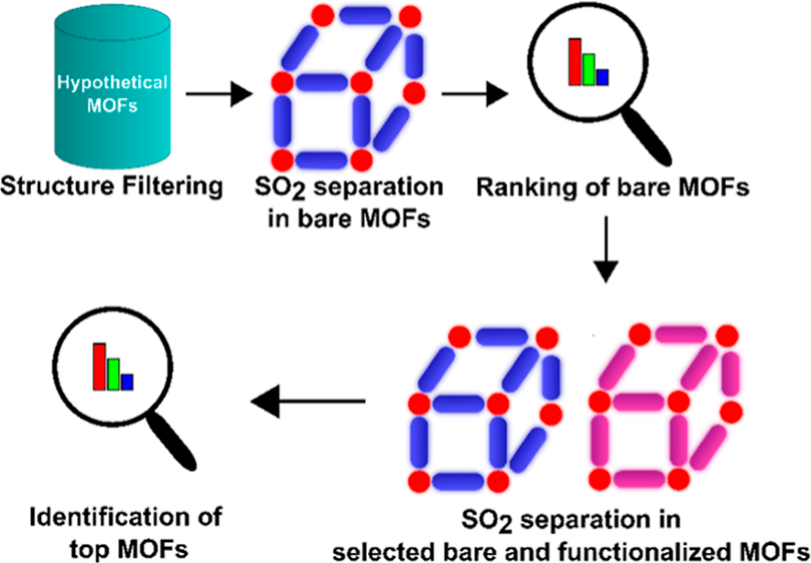

SO_2_ presence in the atmosphere can cause significant harm to the human and environment through acid rain and/or smog formation. Combining the operational advantages of adsorption-based separation and diverse nature of metal–organic frameworks (MOFs), cost-effective separation processes for SO_2_ emissions can be developed. Herein, a large database of hypothetical MOFs composed of >300,000 materials is screened for SO_2_/CH_4_, SO_2_/CO_2_, and SO_2_/N_2_ separations using a multi-level computational approach. Based on a combination of separation performance metrics (adsorption selectivity, working capacity, and regenerability), the best materials and the most common functional groups in those most promising materials are identified for each separation. The top bare MOFs and their functionalized variants are determined to attain SO_2_/CH_4_ selectivities of 62.4–16899.7, SO_2_ working capacities of 0.3–20.1 mol/kg, and SO_2_ regenerabilities of 5.8–98.5%. Regarding SO_2_/CO_2_ separation, they possess SO_2_/CO_2_ selectivities of 13.3–367.2, SO_2_ working capacities of 0.1–17.7 mol/kg, and SO_2_ regenerabilities of 1.9–98.2%. For the SO_2_/N_2_ separation, their SO_2_/N_2_ selectivities, SO_2_ working capacities, and SO_2_ regenerabilities span the ranges of 137.9–67,338.9, 0.4–20.6 mol/kg, and 7.0–98.6%, respectively. Besides, using breakdowns of gas separation performances of MOFs into functional groups, separation performance limits of MOFs based on functional groups are identified where bare MOFs (MOFs with multiple functional groups) tend to show the smallest (largest) spreads.

## 1 Introduction

20th and 21st centuries have witnessed a significant expansion of the industrialization across the globe. Over time, it has been better understood that chemical processes should be run such that the environmental sustainability can be preserved, otherwise, the current global challenges such as air pollution can deteriorate in the future. Currently, the capture of toxic gases from the industrial gas streams and open air is highly critical as the release of acidic gases like SO_2_ into air can lead to acid rain and/or smog, damaging both the human health and environment.^1,2^ It has been reported that in 2018, 62.7 Mt of SO_2_ was emitted to the atmosphere, demonstrating the large extent of the SO_2_ emission problem despite the techniques being used to mitigate the emissions.^3^

Traditionally, the gas separations in the industry are conducted through energy-intensive processes like cryogenic distillation and absorption.^4^ In comparison, adsorption processes can achieve gas separations much more efficiently by taking advantage of sorbate–sorbent interactions around room temperature.^5^ For adsorption processes, there are multiple classes of porous materials that can be implemented. Of them, metal–organic frameworks (MOFs) are highly promising as they are ordered and chemically diverse materials that have been widely investigated especially after 1990s.^6,7^ As their pores can be tailored in terms of size, shape, and functionality, MOFs can offer exceptional gas separation performances with disparate affinities for different adsorbates.^8,9^

In the recent years, more studies started to emerge about the SO_2_ adsorption/separation as a part of efforts to tackle toxic gas problems. As an air pollutant, SO_2_ co-exists with other gases like CH_4_, CO_2_, and N_2_ in the atmosphere^10,11^ where the relative ratios of SO_2_ over other gases can differ considerably depending on the region and operation conditions of the power and/or industrial plants in the region.^12,13^ There are many separation studies where SO_2_ concentrations in binary mixtures vary in a large range of 0.2 to 90%.^3,14−29^ For instance, Zhang et al.^19^ experimentally tested the SO_2_ uptake and separation capability of a Cu-based MOF, CPL-1, under ambient conditions and reported a SO_2_ saturation capacity of 44.8 cm^3^/g, SO_2_/CH_4_ ideal selectivity of 74.3, SO_2_/N_2_ ideal selectivity of 368, and SO_2_/CO_2_ ideal adsorbed solution theory (IAST)^30^ selectivity of 8.7. In a similar experimental work by Zhang et al.,^18^ ELM-12 is reported to have SO_2_/CH_4_, SO_2_/CO_2_, and SO_2_/N_2_ IAST selectivities of 871, 30, and 4064 under ambient conditions for 10% SO_2_ involving mixtures. Considering SO_2_/CO_2_ separation, Brandt et al.^24^ demonstrated that MIL-160 can attain IAST selectivity of ∼125 for a SO_2_/CO_2_ (50/50) mixture around ambient conditions. Similarly, Zhu et al.^25^ estimated the IAST selectivity of MOF-808-His as 90.5 for a SO_2_/CO_2_ (10/90) mixture under ambient conditions. Zárate et al.^31^ synthesized an Al-based MOF (CAU-10) for which experimental (simulated) SO_2_ uptake under ambient conditions is determined to be ∼4.5 (5.2) mol/kg. In another work by Zárate et al.,^22^ MFM-300(Sc) is reported to have an experimental SO_2_ uptake of 9.4 mol/kg under ambient conditions, which agrees with the simulated uptake. Glomb et al.^32^ synthesized an interpenetrated Zn-based MOF functionalized with urea, exhibiting a large SO_2_ uptake of 10.9 mol/kg around ambient conditions.

The expansion of the computational resources and more efficient algorithms have enabled screening the adsorption/separation properties of many materials by which guidance could be provided for the future experimental efforts saving significant time and cost. For instance, Sun et al.^33^ studied 12 porous materials using molecular simulations for the SO_2_ capture from a flue gas mixture and concluded that Cu-BTC and MIL-47 are the best-performing MOFs at 313 K, up to 1 bar in terms of selectivity. Zhang et al.^20^ computationally studied SO_2_ capture from SO_2_/CO_2_ and SO_2_/N_2_ mixtures using porous aromatic frameworks (PAFs) where it has been concluded that the incorporation of the functional groups (−CH_3_, −CN, −COOH, −COOCH_3_, −OH, −OCH_3_, −NH_2_, and −NO_2_) into PAF-1 boosts the SO_2_ selective behavior of the materials especially below 10 bar. Maurya and Singh^34^ simulated the SO_2_ adsorption in several adsorbents (COF-108, COF-300, single-walled carbon nanotube (SWCNT), InOF-1, UiO-66, and ZIF-8) around ambient conditions where the SO_2_ uptakes of UiO-66 and ZIF-8 (∼5 mol/kg) are found to be several folds lower than that of SWCNT (∼23 mol/kg). Li et al.^9^ performed grand canonical Monte Carlo (GCMC) simulations to investigate the SO_2_ capture from SO_2_/CO_2_ and SO_2_/N_2_ mixtures using UiO-66 and its functionalized variants where it has been concluded that UiO-66-(COOH)_2_ and UiO-66-COOH exhibit two of the highest adsorption selectivities at low pressures (SO_2_/CO_2_ and SO_2_/N_2_ selectivity higher than ∼40 and 3000, respectively). As expected, the computational studies focus on higher number of materials than the experimental studies, despite investigating only tens of materials at maximum.

While the sheer number of MOFs implies bigger opportunities, it also necessitates the use of computational tools to expedite the identification of the potentially useful materials. Indeed, it has been previously shown that the computational screening efforts can guide the experimentalists toward the right direction and realize high-performing materials in the laboratory.^35,36^ Motivated by this, a multi-level computational screening study is presented in this work to eventually unlock SO_2_/CH_4_, SO_2_/CO_2_, and SO_2_/N_2_ separation performances of 1770, 2255, and 1909 different MOF materials (filtered from more than 300,000 MOFs), respectively, which constitutes the largest scale computational screening for SO_2_ capture, to the best of our knowledge. In this work, we chose the concentration of SO_2_ in binary mixtures as 10%, which enables the comparison of SO_2_ separation performances of MOFs studied herein with potentially high-performing porous materials probed in many studies^3,16−19,22,24−26,28^ where binary SO_2_/CH_4_, SO_2_/CO_2_, and/or SO_2_/N_2_ mixtures involve 10% SO_2_. While SO_2_ separation from ternary or quaternary mixtures would also be an interesting topic, it is beyond the scope of our work. We first investigate the separation performances of bare hypothetical MOFs and then explore functionalized variants of the top 50 bare materials. Comparing the performances of functionalized and bare MOFs, the most beneficial functional groups are identified for each separation in addition to examining the structure–performance correlations. So far, many studies^37−40^ showed that addition of functional groups improves the separation performances of bare MOFs. Our work demonstrates not only the advantages but also disadvantages of functional group addition using one of the largest functionalized MOF sets.

## 2 Computational Methods

The separation of SO_2_/CH_4_, SO_2_/CO_2_, and SO_2_/N_2_ mixtures (10% SO_2_ content in each) is studied using a hypothetical MOF database^41^ comprising more than 300,000 MOFs. The structures of the database are named as mX_oY_tpl.f where mX (oY) denotes a specific metal (organic) building unit, tpl designates the structure topology, and f represents an internally coded functional group. Thus, a certain f number may not necessarily represent the same functional group in different structures. The porous networks of the structures are analyzed using Zeo++^42,43^ with a probe radius of 1.84 Å to determine global cavity diameter (GCD), pore limiting diameter (PLD), largest cavity diameter (LCD), surface area, probe-occupiable void fraction, and pore volume. All structures investigated in this work were publicly made available on https://archive.materialscloud.org/record/2018.0016/v3.

Mixture gas adsorptions are calculated using GCMC simulations in RASPA.^44^ The GCMC simulations are conducted at two levels where the first one involves the hypothetical MOFs for which no functional group is mentioned (we will refer those materials as bare MOFs), while the second one encompasses both the top 50 bare MOFs and their functionalized variants (those reported with “H” functional group in the database are indeed bare MOFs. Thus, while breaking down the structures into functional groups at the second level, they are collected into “bare” group). The inaccessible pores for sorbates were identified using spheres that are slightly smaller (0.2 Å) than the corresponding sizes of sorbates and blocked via Zeo++ at the first level of the screening. At both levels of GCMC simulations, MOFs whose PLDs are less than sorbate sizes and those with no accessible surface area were excluded. Only structures having no open metal site were investigated (open metal site identification was carried out using Zeo++). In the GCMC simulations, the following moves were allowed with equal probabilities: insertion/deletion, translation, rotation (excluding CH_4_), and identity change. Simulations to determine the gas uptakes were performed at 298 K, 1 (adsorption pressure), and 0.1 (desorption pressure) bar where 20,000 simulation cycles are equally split into equilibration and production cycles. The adsorbate density profiles and radial distribution functions (RDFs) were obtained using 20,000 and 60,000 simulation cycles, respectively, with equal equilibration and production cycles. The interactions of MOF atoms with the gas molecules were defined by universal force field (UFF)^45^ parameters and partial atomic charges in MOFs (PACMOF).^46^ PACMOF charges of hypothetical MOFs were determined using a machine-learning model fitted to density-derived electrostatic and chemical (DDEC) charges (based on 2017 version of the Chargemol package).^147^^47−53^ The sorbate interaction parameters were acquired from earlier studies.^54−56^ The truncation distance for Lennard-Jones interactions was 12 Å. Electrostatic calculations were calculated using the Ewald summation method.^57^ Structures were kept rigid throughout the simulations.

The adsorption selectivity is expressed as , in which *N* represents the adsorbed gas amount obtained from GCMC simulations and *y* is the mole fraction of the gas component in the bulk mixture. Working capacity of a sorbate in a structure is essentially the difference between gas uptakes at the adsorption and desorption conditions (Δ*N*_1_ = *N*_ads,1_ – *N*_des,1_). Regenerability of a structure is defined as . The materials were ranked by the individual gas separation performances (*i.e.*, adsorption selectivity, working capacity, and regenerability), and the overall rankings of materials were determined using the summations of the individual separation performance-based rankings. Thus, the top-ranked materials have the highest overall rankings. We also ranked the materials based on the separation potential (Δ*Q*),^58^ which was calculated as  where *Q*_1_ represents the volumetric uptake capacity of SO_2_.

While SO_2_ may co-exist with H_2_O, it is known that H_2_O adsorption simulations in porous media are typically computationally expensive.^59^ Also, it has been reported that humid SO_2_ exposure can degrade the MOFs considerably while the same MOFs can remain stable after dry SO_2_ exposure.^60,61^ This implies that while GCMC simulations for humid mixtures could have been performed, GCMC results might not describe the gas adsorption/separation behavior of MOFs accurately as the degradation of MOFs is not accounted for in simulations employing rigid frameworks. To eliminate such complexities and keep computational cost at a reasonable level, we simulated dry gas mixtures. To reveal the water affinities of the top structures that we identified, Henry’s constant (*K*_H_) and enthalpy of adsorption (−Δ*H*) for H_2_O (TIP4P model^62^) were calculated at infinite dilution using at least 1,000,000 Widom insertions at 298 K. Adsorbate density profile images were obtained via Paraview.^164^

## 3 Results and Discussion

In our work, we intended to identify the best-performing bare MOFs as the starting platform and probed the functionalized variants thereof to understand which functional group(s) can improve already good performances of bare MOFs. The idea stems from the fact that, in general, it is harder to obtain improvements in separation performances of materials that already perform well. A joint experimental computational work involving about 2 orders of magnitude less number of materials has recently been published.^63^ Therefore, our work focuses on the elucidation of the potential improvement/deterioration in the separation performance of materials due to the grafting of the functional groups on high-performing bare MOFs but not determining separation performances of the entire >300,000 hypothetical MOFs which would be too costly.

Specifically, for all three separations, in the first level of the screening, MOFs, for which no functional group is reported, are filtered from the aforementioned hypothetical MOF database and employed in the GCMC simulations. Using gas separation performance metrics obtained from GCMC simulations, the top performing materials are identified based on the overall rankings, as described above. In the second level of the screening, the top 50 bare MOFs identified in the preceding level and their functionalized variants are utilized in the GCMC simulations from which the top performing hypothetical MOFs are ascertained.

Before we discuss the simulation results, we would like to comment on the choice of UFF. As shown in Table S1, the experimental and simulated SO_2_ uptakes at 1 bar, 298 K in MFM-300(In), and SIFSIX-1-Cu show good agreement (8.28 vs 7.79 mol/kg and 11.01 vs 11.85 mol/kg, respectively). The comparisons of experimental and simulated gas uptakes were based on excess gas uptake values. Helium void fractions, to obtain excess gas uptakes from absolute gas uptakes, were obtained using 10,000,000 Widom insertions at 298 K using the parameters reported earlier.^64^ Having good agreement between experimental and simulated gas uptakes across a certain number of materials would not necessarily guarantee that all simulated gas uptakes would be accurate. This could be due to imperfect experimental crystals (e.g., presence of defects), reproducibility challenges for experimental gas uptakes even in the same material, deficiency of force fields, and/or charge partitioning methods.^65−67^ The main reason to use UFF in the screening studies is that it can predict similar rankings of materials with respect to those obtained by ab initio force fields or experiments, as shown earlier for CO_2_ adsorption, CO_2_/H_2_ selectivity, Xe adsorption, Kr adsorption, and Xe/Kr selectivity.^68−71^ Thus, we employed UFF in this large-scale screening study to obtain trends (e.g., material rankings), and shortlists of promising materials which are more likely to perform better than others.

### 3.1 SO_2_/CH_4_ Separation

Figure S1 illustrates the SO_2_/CH_4_ separation performances of 1295 bare hypothetical MOFs together with their pore features. The top left panel demonstrates that the SO_2_/CH_4_ selectivity, SO_2_ working capacity, and SO_2_ regenerability span the ranges of 3.2–5773.0, 0.1–20.7 mol/kg, and 7.9–98.9%, respectively. While most of the pristine MOFs (992 MOFs) are highly SO_2_ regenerable (>80%), the 10 most SO_2_ selective (over CH_4_) MOFs exhibit low SO_2_ regenerability (8.0–25.2%). The most selective MOFs (selectivity >2000) are those with very narrow pore sizes (4.51–6.88 Å), bringing about significant confinement effects. However, as the narrow pore sizes cause strong interaction potential overlaps at both adsorption and desorption pressures, these structures demonstrate low working capacity and regenerability. Those with the largest SO_2_ working capacities (17.6–20.7 mol/kg) are also largely SO_2_ regenerable (86.7–98.8%), whereas in the low SO_2_ working capacity range (<5 mol/kg), SO_2_ regenerabilities vary in a broad range (7.9–96.2%). The large SO_2_ working capacities are associated with the large-pored structures, in which SO_2_ adsorption is relatively weak at the desorption pressure, leading to not only high working capacity but also high regenerability. In the low working capacity range, SO_2_ selectivities span the entire spectrum (3.2–5773.0), suggesting that significant trade-offs can be seen across selectivity and working capacity. There is a branch in the plot (selectivity < 20 and working capacity < 5 mol/kg) where selectivities drop with the decrease in the working capacity. This region involves structures with highly varying PLDs (6.59–21.89 Å) where the SO_2_ uptakes at the adsorption pressure span a narrow range of 0.1–1.1 mol/kg, accompanied with a narrow SO_2_ regenerability range (85.9–92.8%).

The top right panel relates the SO_2_/CH_4_ selectivity and SO_2_ working capacity with the porosity (*i.e.*, void fraction) of the structures. Not surprisingly, large SO_2_ working capacities (>10 mol/kg) are predicted for some of the vastly porous structures (void fraction >0.7); however, those with the highest void fractions (0.881–0.919) are found to attain very limited SO_2_ working capacities (<2 mol/kg), implying that there is not a straightforward relation between SO_2_ working capacity and void fraction. The selectivity *versus* PLD relation in the bottom left panel demonstrates that the most SO_2_ selective (over CH_4_) structures are those having narrow pore sizes. However, around small PLD values (5–6 Å), SO_2_/CH_4_ selectivities extend in a large range (21.3–5773.0), implying that a material screening solely based on PLD values would not result in a shortlist of materials with only high selectivities. Similarly, especially for small PLDs (<6 Å), the void fractions can vary greatly (0.166–0.744), signifying the diverse structural properties of the structures. As the pore sizes expand, structures lose their SO_2_ selective (over CH_4_) behavior significantly with the lowest SO_2_/CH_4_ selectivity of 3.2 at a PLD of 13.30 Å. The bottom right panel depicts that the most SO_2_ selective (over CH_4_) structures are populated in a wide surface area spectrum (1325.4–3360.5 m^2^/g), which resembles a peak as selectivities are lower at smaller and larger surface area values. However, it is also seen that there are many other MOFs with similar surface area values with significantly less selective behavior.

**Figure 1:**
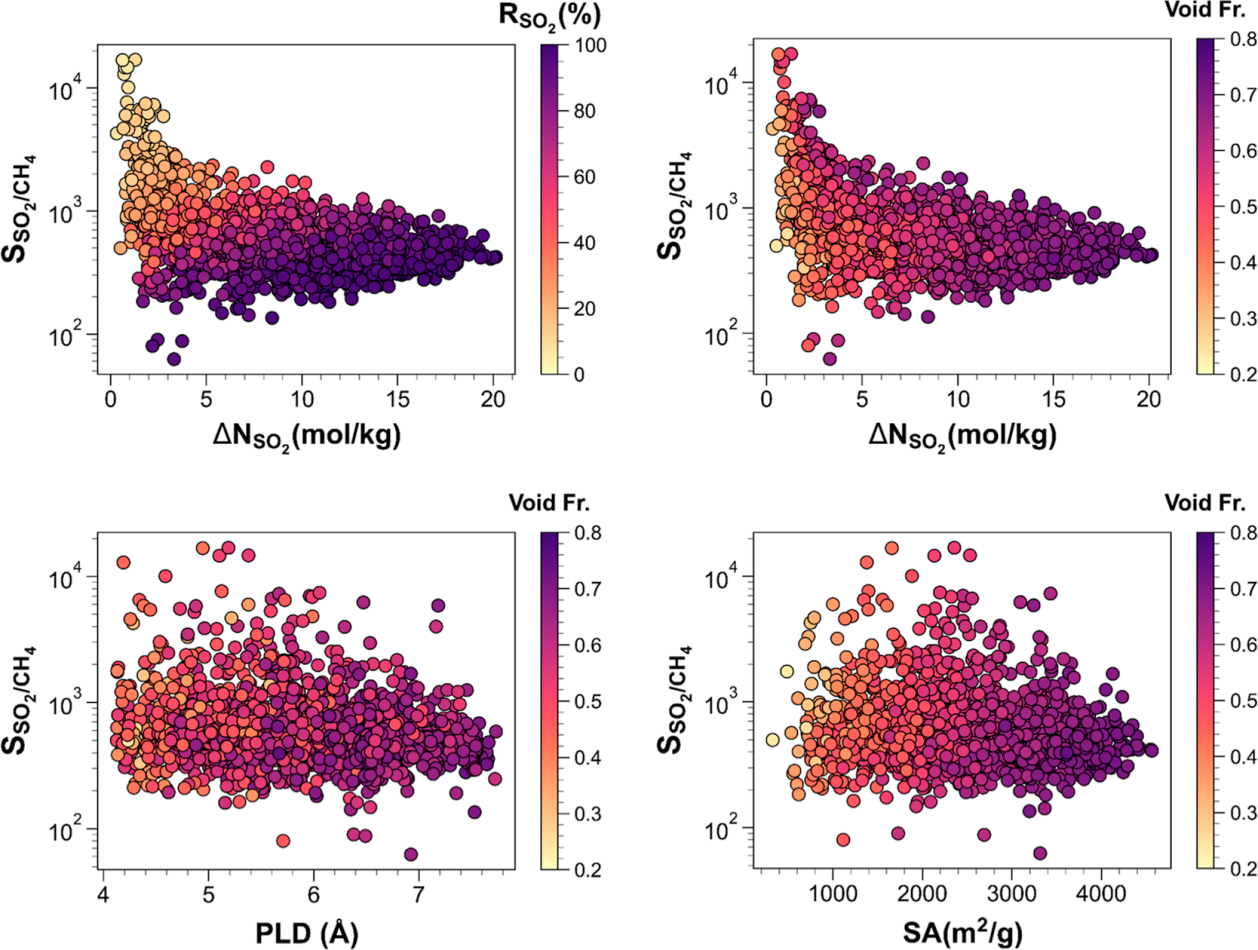
SO_2_/CH_4_ separation performances of the top 50 bare MOFs and their functionalized counterparts along with their textural features.

Figure 1 demonstrates the SO_2_/CH_4_ separation performances of the top 50 bare MOFs and their functionalized variants (functional groups are −F, −Cl, −Br, −I, −Me (−CH_3_), −Et (−CH_2_CH_3_), −Pr (−CH_2_CH_2_CH_3_), −HCO, −COOH, −OH, −OMe, −OEt, −OPr, −NH_2_, −CN, −NHMe, −NO_2_, −Ph (Ph = phenyl), −SO_3_H) as well as their pore features. The top left panel portrays the large extents of SO_2_/CH_4_ selectivity (62.4–16,899.7), SO_2_ working capacity (0.3–20.1 mol/kg), and SO_2_ regenerability (5.8–98.5%). It also reveals the inverse relations of SO_2_/CH_4_ selectivity versus SO_2_ working capacity and SO_2_/CH_4_ selectivity versus SO_2_ regenerability. For instance, a functionalized MOF, m2_o12_o29_pcu.199 (having multiple functional groups of −NO_2_ and −NH_2_) with the highest SO_2_/CH_4_ selectivity of 16899.7 is deprived of large SO_2_ working capacity and regenerability (being only 1.3 mol/kg and 9.9%, respectively). On the other hand, a bare MOF, m2_o12_o27_pcu.106 attaining the largest SO_2_ working capacity of 20.1 mol/kg and a high SO_2_ regenerability of 96.9%, has a large SO_2_/CH_4_ selectivity of 423.3. The top right panel demonstrates that MOFs with large SO_2_ working capacities (>10 mol/kg) possess medium–high void fractions (0.517–0.744). However, some of the structures with moderate porosities (0.4–0.7) can also attain low SO_2_ working capacities down to 0.6 mol/kg, suggesting that MOFs with moderate-high porosities cover a wide SO_2_ working capacity spectrum. A similar conclusion can be made for the SO_2_/CH_4_ selectivities of those structures (62.4–16,899.7). The bottom left panel illustrates that the 10 most SO_2_ selective (over CH_4_) structures possess PLDs in the limits of 4.19–6.05 Å, in which the top four selective structures are in a very narrow range of 4.94–5.38 Å, suggesting strong confinement effects. The bottom right panel depicts that the five most SO_2_ selective (over CH_4_) MOFs possess surface areas between 1378.4 and 2531.4 m^2^/g, while those next to them have lower SO_2_/CH_4_ selectivities giving rise to a peak around 2000 m^2^/g. It also portrays the large extents of selectivities (89.9–16,792.9) in a narrow surface area range (1600–1800 m^2^/g), which is not surprising as selectivities are governed by multiple factors such as pore size, shape, porosity, functional group, and so forth.

**Figure 2:**
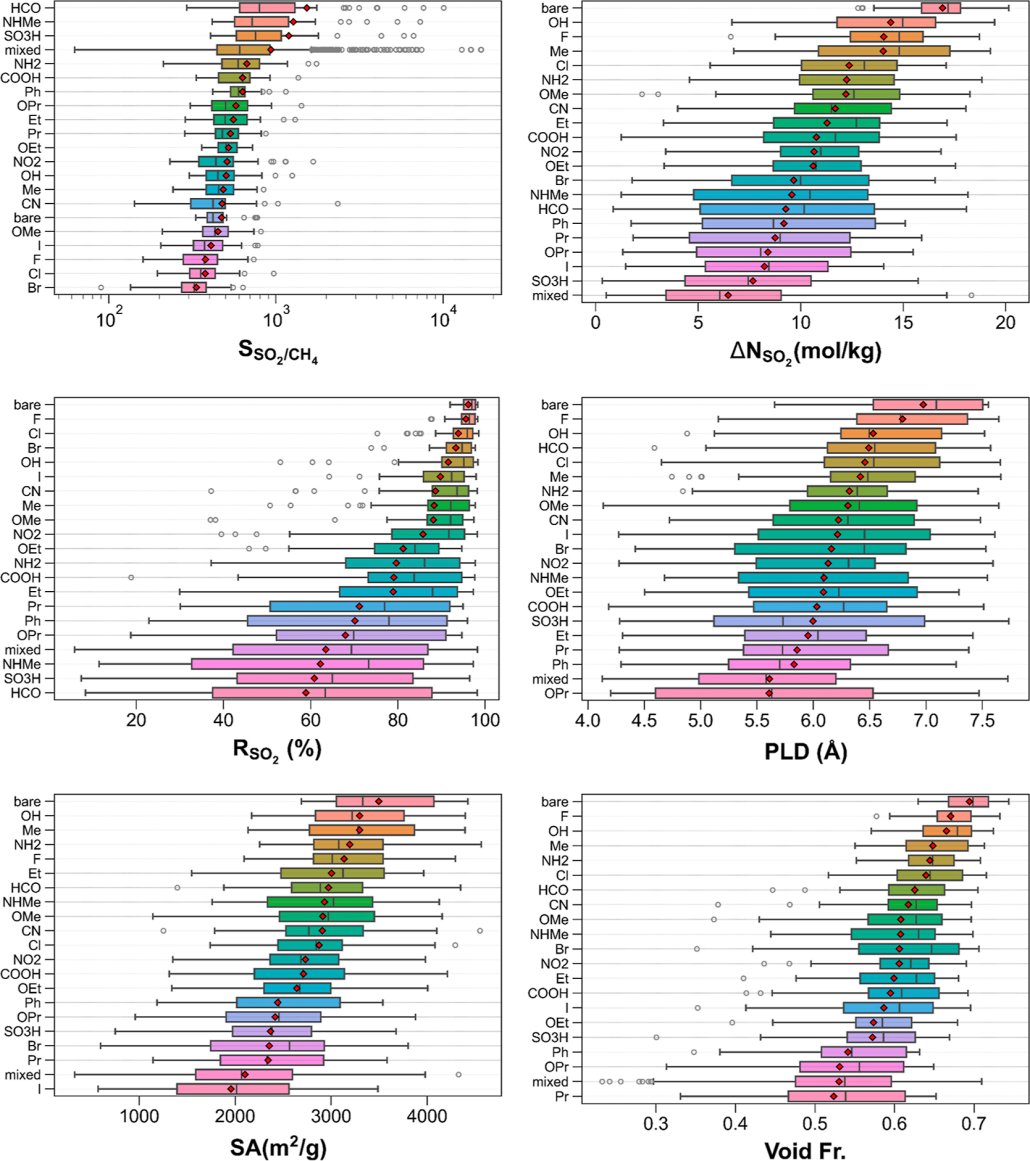
SO_2_/CH_4_ separation performances and structural properties of the top 50 bare MOFs and their functionalized counterparts classified by their functional groups (note the following for all box-and-whisker plots: mean values are denoted with red diamonds. Subgroups of MOFs are sorted from top to bottom by mean values in the descending order. Boxes denote the range of values from the first quartile to the third quartile, and whiskers demonstrate the rest of the distribution except outliers. Outliers (shown as empty circles) are values that are away from either end of the box by more than 1.5 interquartile range. Boxes are colored only to guide the eye for differentiating different groups of MOFs; box colors are not based on any material property).

Figure 2 delineates the breakdown of SO_2_/CH_4_ separation performances and structural features of the top 50 bare MOFs and their functionalized variants into functional groups. The top left panel shows that MOFs with −HCO, −NHMe, and SO_3_H functional groups are generally more SO_2_ selective (over CH_4_) than other monofunctionalized MOFs. On average, the lowest SO_2_/CH_4_ selectivities are predicted for halogen (−I, −F, −Cl, and −Br)-functionalized MOFs, which are less selective than bare MOFs. MOFs with multiple functional groups (“mixed” case) demonstrate the largest spread in SO_2_/CH_4_ selectivities, which is expected as they have about 300 subgroups (e.g., −OH–Cl, −COOH–NH_2_, *etc.*). While very low SO_2_/CH_4_ selectivities (less than 100) can be found in this group, their mean SO_2_/CH_4_ selectivities are fourth largest following −HCO, −NHMe, and −SO_3_H. In addition, this group involves the most SO_2_ selective (over CH_4_) MOFs with −NO_2_–NH_2_, −NHMe–HCO, and −HCO–OEt groups in the top three selective MOFs.

The top right panel illustrates the SO_2_ working capacities of the MOFs categorized by their functional groups where bare MOFs attain larger SO_2_ working capacities than the functionalized MOFs while those with multiple functional groups exhibit the lowest SO_2_ working capacities on average. However, the spread of SO_2_ working capacities for the latter is very broad (starting around 0 and going beyond 15 mol/kg), implying that there are MOFs with multiple functional groups that can attain similar SO_2_ working capacities to those of monofunctionalized MOFs. Among the monofunctionalized MOFs, those with −OH, −F, and −Me groups achieve the biggest SO_2_ working capacities on average. Looking at the other end of the spectrum, MOFs with −SO_3_H and −I functional groups exhibit the second and third least SO_2_ working capacities following MOFs with multiple functional groups. The middle-left panel illustrates SO_2_ regenerabilities of the MOFs grouped by the functional groups where bare MOFs exhibit the highest SO_2_ regenerabilities on average and the smallest spread. Among functionalized MOFs, MOFs functionalized with three types of halogens (−F, −Cl, and −Br) have the highest SO_2_ regenerabilities, while those functionalized with −HCO, −SO_3_H, and −NHMe show the smallest SO_2_ regenerabilities on average. MOFs with multiple functional groups demonstrate the fourth lowest mean SO_2_ regenerabilities and the widest spread (*R*_SO_2__ = ∼6–98%), which is comparable to those of MOFs with -NHMe, −SO_3_H, and −HCO functional groups.

The middle-right panel portrays the extents of PLDs of MOFs categorized by functional groups. Overall, bare MOFs tend to have larger PLDs than functionalized MOFs, which is expected as the functional groups, can reduce the available pore space along the path where PLD is located. There are some cases where the functionalized MOFs have slightly larger PLDs than the functionalized MOFs, which can be attributed to the structure optimization, leading to more open structures. As the functional groups grow, average PLDs diminish, as exemplified by MOFs with −OPr, −Ph, and −Pr groups. Another case where average PLDs are lower than those of bare MOFs is MOFs with multiple functional groups, which is not surprising as the incorporation of multiple functional groups can decrease the pore sizes more dramatically than monofunctionalized MOFs, especially when the monofunctional group is small. The bottom left panel demonstrates the surface areas of the MOFs categorized by the functional groups. Bare MOFs possess the highest surface areas, whereas those functionalized with −I group have the lowest surface areas on average. Among the functionalized MOFs, those with −OH, −Me, and −NH_2_ groups have the largest mean surface areas. The bottom right panel displays the void fractions of the MOFs classified by the functional groups. Similar to the observations made for PLDs and surface areas, bare MOFs exhibit the largest mean void fractions. Among the functionalized MOFs, MOFs with −F, −OH, and −Me groups have the highest void fractions overall. The smallest average void fractions are demonstrated by MOFs with bulky groups (−Pr and −OPr) and multiple functional groups.

### 3.2 SO_2_/CO_2_ Separation

Figure S2 depicts the SO_2_/CO_2_ separation performance metrics and structural features of 1295 bare MOFs where the SO_2_/CO_2_ selectivity, SO_2_ working capacity, and SO_2_ regenerability span the ranges of 2.3–372.1, 0.1–19.4 mol/kg, and 6.6–98.7%, respectively. The top left panel shows that most of the bare MOFs possess high SO_2_ regenerability (>80%) covering a SO_2_/CO_2_ selectivity range of 2.3–86.9 and the entire SO_2_ working capacity spectrum. The most notable examples of MOFs lacking good SO_2_ regenerabilities are those with high SO_2_/CO_2_ selectivities. The top right panel reveals that bare MOFs with limited SO_2_ working capacities (<5 mol/kg) can have a wide variety of void fractions (0.166–0.919). Notably, the most porous bare MOFs (void fraction > 0.9) exhibit very low SO_2_ working capacities (<2 mol/kg). The bottom left panel exhibits the inverse relation between the SO_2_/CO_2_ selectivity and PLDs of bare MOFs where at narrow PLD sizes (<6 Å), both low and high SO_2_/CO_2_ selectivities are obtained with void fractions covering a wide range of 0.166–0.744. The most (least) SO_2_ selective bare MOF is m3_o10_o25_pcu.1 (m1_o20_o20_pcu.1) with SO_2_/CO_2_ selectivity of 372.1 (2.3) and PLD of 5.05 (13.30) Å. The bottom right panel unravels the fact that the most SO_2_ selective (over CO_2_) bare MOFs can have highly varying surface areas of 562.4–3360.5 m^2^/g.

Figure S3 illustrates the SO_2_/CO_2_ separation performance metrics and textural properties of the top 50 performing bare MOFs and their functionalized variants. As the top left panel shows, the most SO_2_ selective MOFs have very limited working capacities and regenerabilities. In general, MOFs having high SO_2_ working capacities also possess large regenerabilities, as exemplified by m2_o12_o27_pcu.138 having the largest SO_2_ working capacity of 17.7 mol/kg and a high SO_2_ regenerability of 96.5%. The top right panel shows that going from low to high SO_2_ working capacities, void fractions generally increase. This implies that the SO_2_ adsorption at the desorption pressure in MOFs with high void fractions remains at relatively low values while that at the adsorption pressure can attain much larger values than those in MOFs with limited void fractions despite some exceptions. Considering the SO_2_/CO_2_ selectivity, PLD, and void fraction correlations in the bottom left panel, it can be deduced that at a particular PLD value, widely varying SO_2_/CO_2_ selectivities and void fractions can be obtained. The bottom right panel shows that the most SO_2_ selective MOFs are not the ones with very low or high surface areas but instead moderate surface areas.

**Figure 3:**
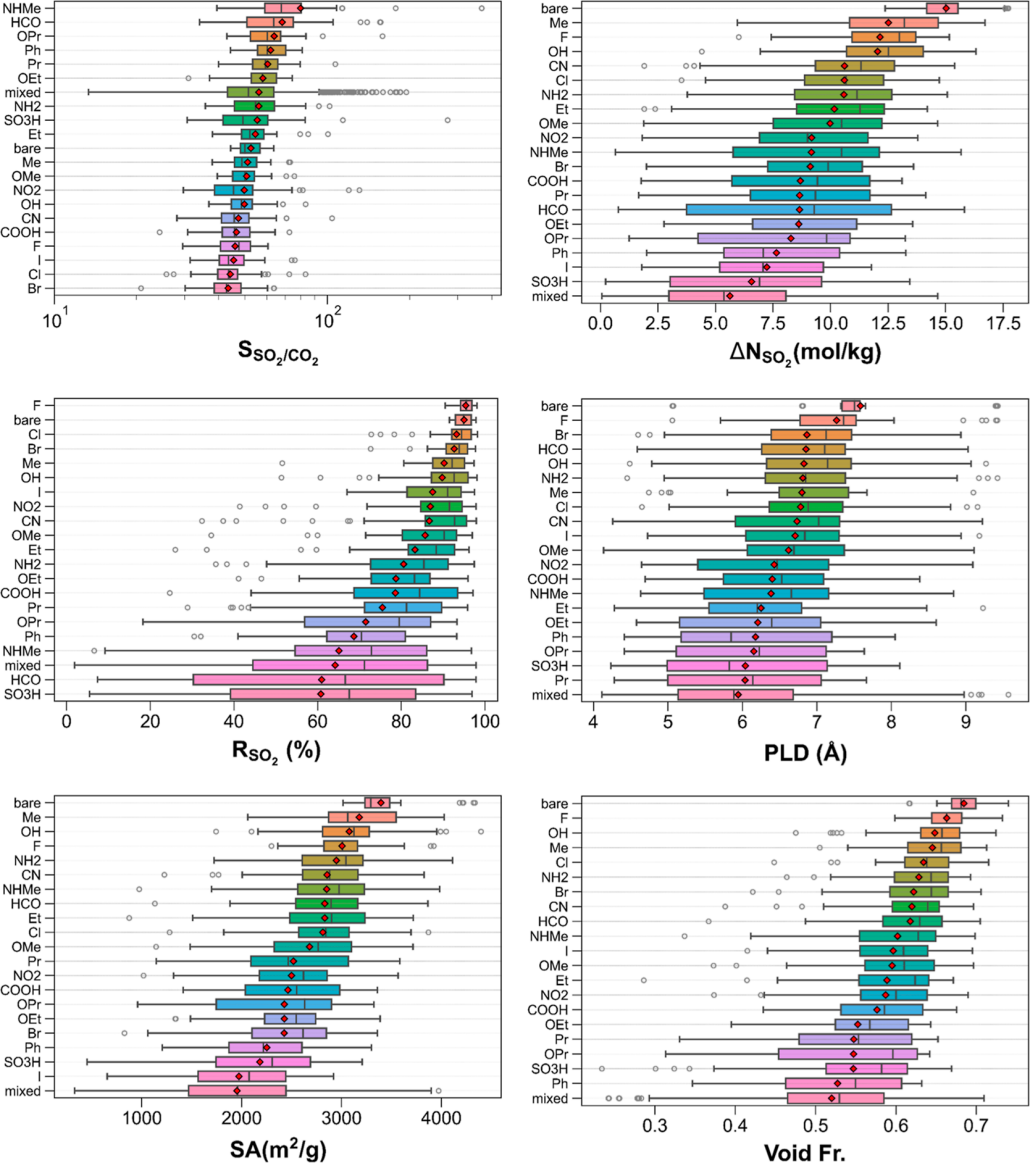
SO_2_/CO_2_ separation performances and structural features of the top 50 bare MOFs and their functionalized counterparts categorized by their functional groups.

Figure 3 portrays the SO_2_/CO_2_ separation performance metrics and pore features of the 50 best-performing bare MOFs and their functionalized counterparts. Comparing the ranges of selectivities of bare and functionalized MOFs in the top left panel, it can be concluded that the incorporation of the functional groups can considerably expand the limits of SO_2_/CO_2_ selectivities of MOFs. On average, MOFs with the functional groups of −NHMe, −HCO, and −OPr exhibit the largest SO_2_/CO_2_ selectivities while those functionalized with halogen groups (−F, −I, −Cl, and −Br) are the least selective. Bare MOFs stand in the middle of the boxplot in terms of mean SO_2_/CO_2_ selectivities, suggesting that depending on the type of functional groups, MOF functionalization can lead to higher or lower mean SO_2_/CO_2_ selectivities. Some MOFs with multiple functional groups attain the lowest SO_2_/CO_2_ selectivities (*e.g.*, m3_o13_o24_pcu.240 functionalized with −Br and −HCO groups having the smallest SO_2_/CO_2_ selectivity of 13.3), indicating that multiple functional groups can be less beneficial for selectivity than single type of functional groups. The top right panel demonstrates that bare MOFs tend to show higher SO_2_ working capacities than the functionalized MOFs. Among the functionalized MOFs, those with −Me, −F, and −OH groups attain the largest SO_2_ working capacities on average. MOFs with multiple functional groups tend to show the lowest SO_2_ working capacities, as evidenced by their smallest mean and median SO_2_ working capacities. However, they also exhibit one of the largest spreads in SO_2_ working capacity (14.6 mol/kg), in which the smallest (largest) SO_2_ working capacity of 0.1 (14.7) mol/kg is attained by a MOF functionalized with −Br and −HCO (−Cl and −F) groups. All in all, these distributions hint that adjusting the gas affinities of MOFs through functionalization may not necessarily lead to higher SO_2_ working capacities than those of bare MOFs as the pore spaces typically decrease via functionalization, and the gas uptake could be strong not only at the adsorption pressure but also at desorption pressure.

The middle-left panel depicts that the MOFs with three different halogen (−F, −Cl, and −Br) groups and bare MOFs demonstrate the largest SO_2_ regenerabilities with comparable mean SO_2_ regenerabilities (around 93–95%) and small spreads. It can be inferred that MOF functionalization can bring about significant reductions in SO_2_ regenerabilities, especially when there are multiple functional groups and/or relatively large functional groups. The middle-right panel illustrates that, on average, functionalized MOFs have smaller PLDs than bare MOFs where the lowest mean PLDs belong to MOFs with multiple functional groups followed by MOFs with −Pr and −SO_3_H groups. Among the functionalized MOFs, the largest mean PLDs are exhibited by MOFs with −F, −Br, and −HCO groups. The bottom left panel delineates the surface area distributions of MOFs categorized by their functional groups where the largest mean surface areas are exhibited by bare MOFs followed by the MOFs functionalized with −Me and −OH groups. MOFs with multiple functional groups possess the smallest surface areas among which m3_o12_o17_pcu.187 (functionalized with −Pr and −I groups) has the lowest surface area of 327.5 m^2^/g. However, as there are about 300 subgroups in mixed functional group cases, MOFs with multiple functional groups show the largest spread in surface area extending up to 3971.3 m^2^/g. It has been observed that large functional groups such as −I, −SO_3_H, and −Ph can drastically diminish the surface area of bare MOFs. Similar conclusions can be made for void fractions, as shown in the bottom right panel where the lowest mean void fractions are attained by MOFs with multiple functional groups and MOFs with large functional groups (−Ph, −SO_3_H, and −OPr).

### 3.3 SO_2_/N_2_ Separation

Figure S4 manifests the SO_2_/N_2_ separation performances of 1295 bare MOFs along with textural properties thereof. The top left panel demonstrates that the 10 most SO_2_ selective (over N_2_) MOFs (SO_2_/N_2_ selectivity of 14,418.6–28,768.3) suffer from relatively low SO_2_ regenerabilities (<25%), whereas most of the remaining MOFs exhibit high SO_2_ regenerabilities (>80%). The top right panel demonstrates that the 20 most SO_2_ selective structures (SO_2_/N_2_ selectivity of 8433.0–28,768.3) possess widely varying void fractions (0.310–0.699). Considering the top left and top right panels, it can be deduced that MOFs exhibiting highly favorable separation performances in terms of all three metrics possess medium–high void fractions (>0.65). The bottom left panel illustrates that some of the screened MOFs show vastly different selectivities despite having similar PLDs (e.g., MOFs with PLDs of 12.5–13.5 Å attain SO_2_/N_2_ selectivities of 4.3–2275.7). A similar conclusion can be drawn from the bottom right panel as the MOFs with similar surface areas (e.g., 3300–3400 m^2^/g) can acquire highly dissimilar SO_2_/N_2_ selectivities (14.7–28,768.3).

Figure S5 depicts the SO_2_/N_2_ separation performance metrics and structural properties of the 50 top performing bare MOFs and functionalized versions thereof. In the top left panel, SO_2_/N_2_ selectivities reach up to 67,338.9 where the most SO_2_ selective (over N_2_) MOFs are functionalized MOFs (m2_o12_o29_pcu.213 being the most SO_2_ selective). Generally, MOFs with high SO_2_ working capacities (>10 mol/kg) show high SO_2_ regenerabilities (>80%) as well. The top right panel shows that highly porous (void fraction > 0.7) MOFs attain large SO_2_ working capacities (>10 mol/kg). As expected, MOFs with low void fractions (<0.3) demonstrate limited SO_2_ working capacities (0.6–2.0 mol/kg). The bottom left panel depicts that high SO_2_/N_2_ selectivities (>10^4^) can be obtained in MOFs with PLDs varying in a wide range (4.16–8.65 Å), whereas the least selective MOFs (SO_2_/N_2_ selectivity < 300) are seen between PLDs of 5.71 and 6.92 Å. The bottom right panel reveals that prescreening materials for high selectivity solely based on surface area would not be ideal as the SO_2_/N_2_ selectivities of MOFs with similar surface areas can be drastically different.

**Figure 4:**
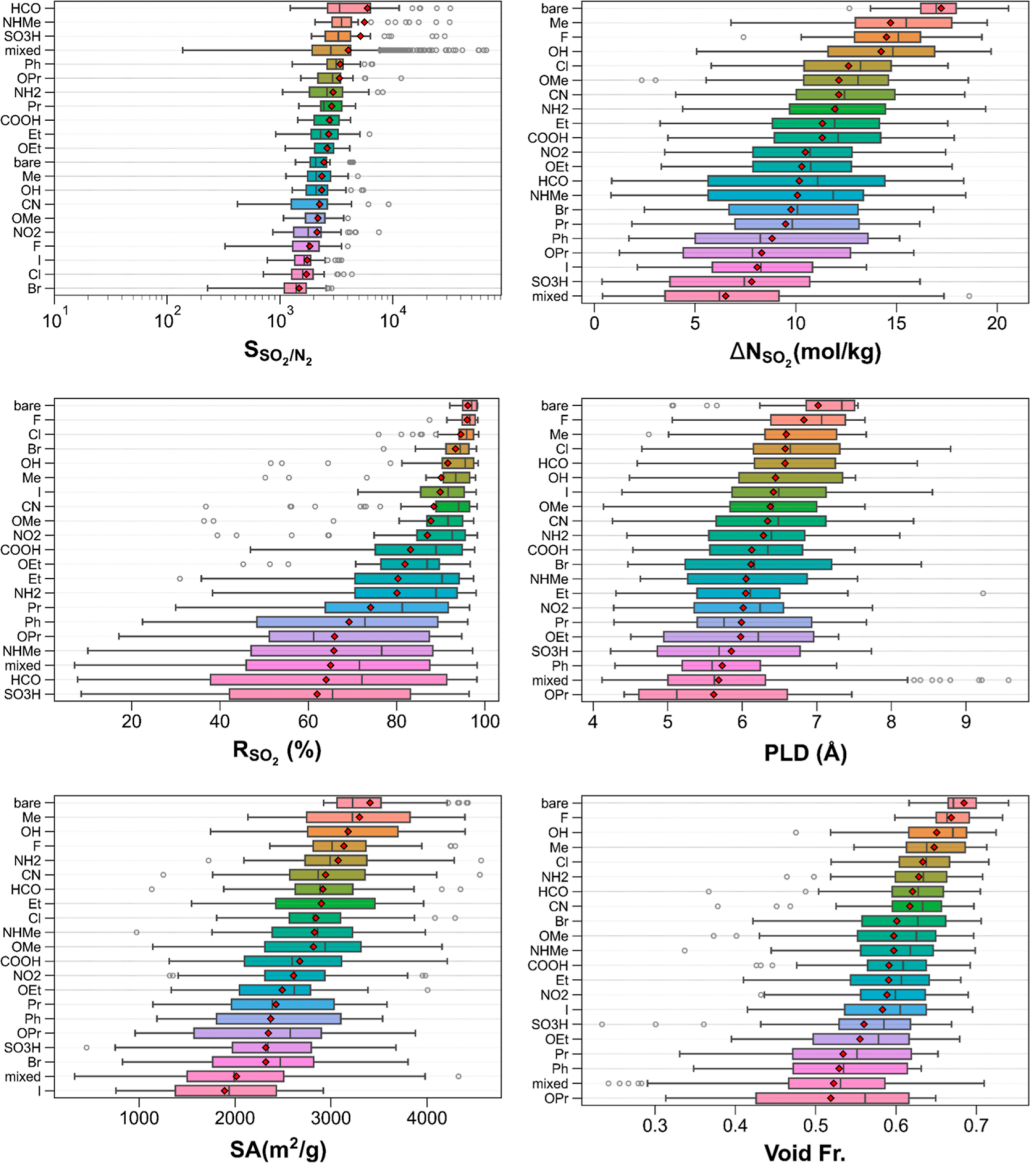
SO_2_/N_2_ separation performances and structural properties of the top 50 bare MOFs and their functionalized counterparts grouped by their functional groups.

Figure 4 delineates the SO_2_/N_2_ separation performances and textural features of the 50 best bare MOFs and their corresponding functionalized variants. The top left panel unravels that on average MOFs with −HCO, −NHMe, and −SO_3_H functional groups are more SO_2_ selective (over N_2_) than the others. However, the most diverse group is MOFs with multiple functional groups, demonstrating the widest range of SO_2_/N_2_ selectivities (137.9–67338.9). Among them, m2_o12_o29_pcu.213 is the most SO_2_ selective (over N_2_) MOF having the functional groups of −HCO and −OEt. There are nine groups of MOFs (functionalized with −Me, −OH, −CN, −OMe, −NO_2_, −F, −I, −Cl, and −Br) that have lower mean SO_2_/N_2_ selectivities than that of bare MOFs, indicating that MOF functionalization does not necessarily render a MOF more selective. The top right panel unveils that bare MOFs show the largest mean SO_2_ working capacity of 17.2 mol/kg (followed by MOFs monofunctionalized with relatively small groups, namely, −Me and −F), whereas MOFs with multiple functional groups attain the smallest mean SO_2_ working capacity of 6.5 mol/kg. Despite the low average SO_2_ working capacity, the latter group acquires the widest range of SO_2_ working capacity (0.4–18.6 mol/kg), hinting that the loss of pore space through multiple functionalization does not necessarily bring about a low SO_2_ working capacity. Another observation is that MOFs with bulky functional groups such as −OPr, −I, and −SO_3_H attain some of the smallest mean SO_2_ working capacities after MOFs with multiple functional groups, albeit having a large spectrum of SO_2_ working capacity values. The middle-left panel reveals that bare MOFs and MOFs monofunctionalized with halogen groups (*i.e.*, −F, −Cl, and −Br) exhibit the largest SO_2_ regenerabilities overall (mean SO_2_ regenerabilities around 93–96%). On the contrary, MOFs monofunctionalized with −SO_3_H and −HCO groups and MOFs with multiple functional groups are the least SO_2_ regenerable structures on average (SO_2_ regenerabilities around 62–65%). The middle-right panel suggests that while bare MOFs have the largest PLDs on average, compared to bare MOFs, some of the functionalized MOFs can possess higher PLDs (>7.3 Å), which can be ascribed to linker rotation because of structure optimization following functionalization. It is crucial to note this counter-intuitive observation as PLDs would typically be anticipated to decrease as the functionalization may narrow down the pore aperture especially when the functional groups are oriented toward the pore center. The bottom left and right panels show that the bare MOFs tend to have the largest surface areas and void fractions while those with large (−I, −OPr *etc.*) or multiple functional groups possess the smallest surface areas and void fractions overall.

Table 1 tabulates the top 10 MOFs determined for each separation, which are ranked by their overall ranking, as defined above. Among the top MOFs for SO_2_/CH_4_ separation, all MOFs are Cu-based functionalized MOFs. The top ranked MOF, m2_o12_o29_pcu.260 (functional groups = −OH–Cl), is the only MOF with multiple functional groups in the top 10 MOF list. Among the 10 best MOFs for the SO_2_/CO_2_ separation, one MOF is Zn-based while the rest is Cu-based. Four MOFs among the top 10 list are bare MOFs while two MOFs have multiple functional groups (−Me–Cl and −F–HCO). In the top 10 list, while there are more SO_2_ selective structures than the top three MOFs, they possess smaller SO_2_ working capacities and regenerabilities rendering their overall rankings lower (*e.g.*, 6th and 8th). All the top 10 hypothetical MOFs determined for the SO_2_/N_2_ separation are Cu-based MOFs. In them, there are six bare MOFs, three monofunctionalized MOFs, and one MOF with multiple functional groups (−OH–Cl). The best three MOFs for SO_2_/N_2_ separation are m2_o12_o18_pcu.79 (−CN functionalized), m2_o12_o29_pcu.249 (−OH functionalized), and m2_o12_o29_pcu.260 (−OH–Cl functionalized).

**Table 1:** Top 10 MOFs Identified (Based on Overall Rankings) for the SO_2_/CH_4_, SO_2_/CO_2_, and SO_2_/N_2_ Separation^a^

structure	*S*_SO_2_/X_	Δ*N*_SO_2__ (mol/kg)	*R*_SO_2__ (%)	GCD (Å)	PLD (Å)	LCD (Å)	SA (m^2^/g)	void fr.	pore vol. (cm^3^/g)	func. group	metal
SO_2_/CH_4_											
m2_o12_o29_pcu.260	669.8	18.3	96.0	7.63	5.63	7.58	3979.3	0.695	1.115	OH–Cl	Cu
m2_o12_o29_pcu.221	630.4	19.5	96.3	7.74	6.38	7.74	4202.2	0.711	1.209	OH	Cu
m2_o12_o27_pcu.188	636.1	18.0	95.7	8.43	6.92	8.38	3731.9	0.680	1.129	NHMe	Cu
m2_o12_o29_pcu.2	642.1	17.4	95.7	7.70	6.66	7.69	3819.1	0.685	1.088	OH	Cu
m2_o12_o27_pcu.168	575.4	18.1	97.3	7.66	6.87	7.63	3511.5	0.686	1.137	NHMe	Cu
m2_o12_o29_pcu.249	684.1	16.8	95.2	7.62	6.36	7.62	3674.3	0.672	1.035	OH	Cu
m2_o12_o29_pcu.85	555.7	19.0	97.6	7.75	6.37	7.74	4175.3	0.709	1.206	OH	Cu
m2_o12_o29_pcu.155	531.3	19.1	97.5	7.83	6.65	7.83	4212.5	0.707	1.195	OH	Cu
m2_o12_o29_pcu.157	685.0	17.6	94.0	7.60	6.34	7.60	4056.3	0.692	1.112	COOH	Cu
m2_o12_o18_pcu.79	766.7	14.8	94.4	7.79	6.49	7.70	2590.2	0.617	0.846	CN	Cu
SO_2_/CO_2_											
m2_o11_o17_pcu.118	62.1	15.3	94.6	9.17	7.65	9.17	3054.3	0.686	1.060	Me	Cu
m2_o11_o17_pcu.143	61.2	14.6	94.3	8.64	7.42	8.57	3013.6	0.665	1.005	Me	Cu
m2_o11_o17_pcu.95	62.7	15.6	93.2	9.16	7.65	9.13	3240.8	0.700	1.102	bare	Cu
m2_o17_o22_pcu.190	62.5	16.1	92.9	9.96	7.45	9.95	3421.6	0.668	1.094	bare	Cu
m2_o17_o22_pcu.29	63.0	16.1	92.6	9.96	7.45	9.96	3491.9	0.669	1.096	bare	Cu
m3_o12_o17_pcu.56	75.5	13.4	91.0	7.83	7.36	7.72	2708.6	0.641	0.881	Me–Cl	Zn
m2_o11_o17_pcu.246	63.6	13.6	93.5	9.15	7.65	9.15	2748.1	0.680	0.950	Br	Cu
m2_o11_o17_pcu.106	74.7	12.5	92.5	8.12	7.45	8.12	2495.7	0.628	0.812	F–HCO	Cu
m2_o17_o22_pcu.257	62.2	16.2	92.6	9.95	7.45	9.95	3510.6	0.669	1.096	bare	Cu
m2_o11_o17_pcu.265	58.7	14.5	94.2	9.04	7.65	9.04	3032.5	0.679	1.024	HCO	Cu
SO_2_/N_2_											
m2_o12_o18_pcu.79	4314.6	15.0	94.7	7.79	6.49	7.70	2590.2	0.617	0.846	CN	Cu
m2_o12_o29_pcu.249	3396.7	17.1	95.3	7.62	6.36	7.62	3674.3	0.672	1.035	OH	Cu
m2_o12_o29_pcu.260	3114.0	18.6	95.8	7.63	5.63	7.58	3979.3	0.695	1.115	OH–Cl	Cu
m2_o12_o18_pcu.84	4380.1	16.4	92.5	7.86	6.86	7.67	3085.9	0.665	0.989	bare	Cu
m2_o12_o18_pcu.195	4461.8	16.4	92.1	7.88	6.85	7.65	3068.7	0.665	0.988	bare	Cu
m2_o12_o18_pcu.162	3278.0	14.7	97.1	7.80	6.46	7.80	2544.3	0.628	0.839	Cl	Cu
m2_o12_o18_pcu.87	4186.6	16.4	92.7	7.86	6.86	7.73	3079.8	0.665	0.989	bare	Cu
m2_o12_o18_pcu.46	4218.7	16.4	92.4	7.88	6.85	7.65	3055.0	0.665	0.988	bare	Cu
m2_o12_o18_pcu.4	4168.8	16.4	92.5	7.86	6.86	7.73	3053.3	0.665	0.989	bare	Cu
m2_o12_o18_pcu.42	4292.3	16.4	92.0	7.86	6.86	7.73	3048.7	0.665	0.989	bare	Cu

a GCD = global cavity diameter, PLD = pore limiting diameter, LCD = largest cavity diameter, SA = surface area, void fr. = void fraction, pore vol. = pore volume, and X = CH_4_, CO_2_, or N_2_.

**Figure 5:**
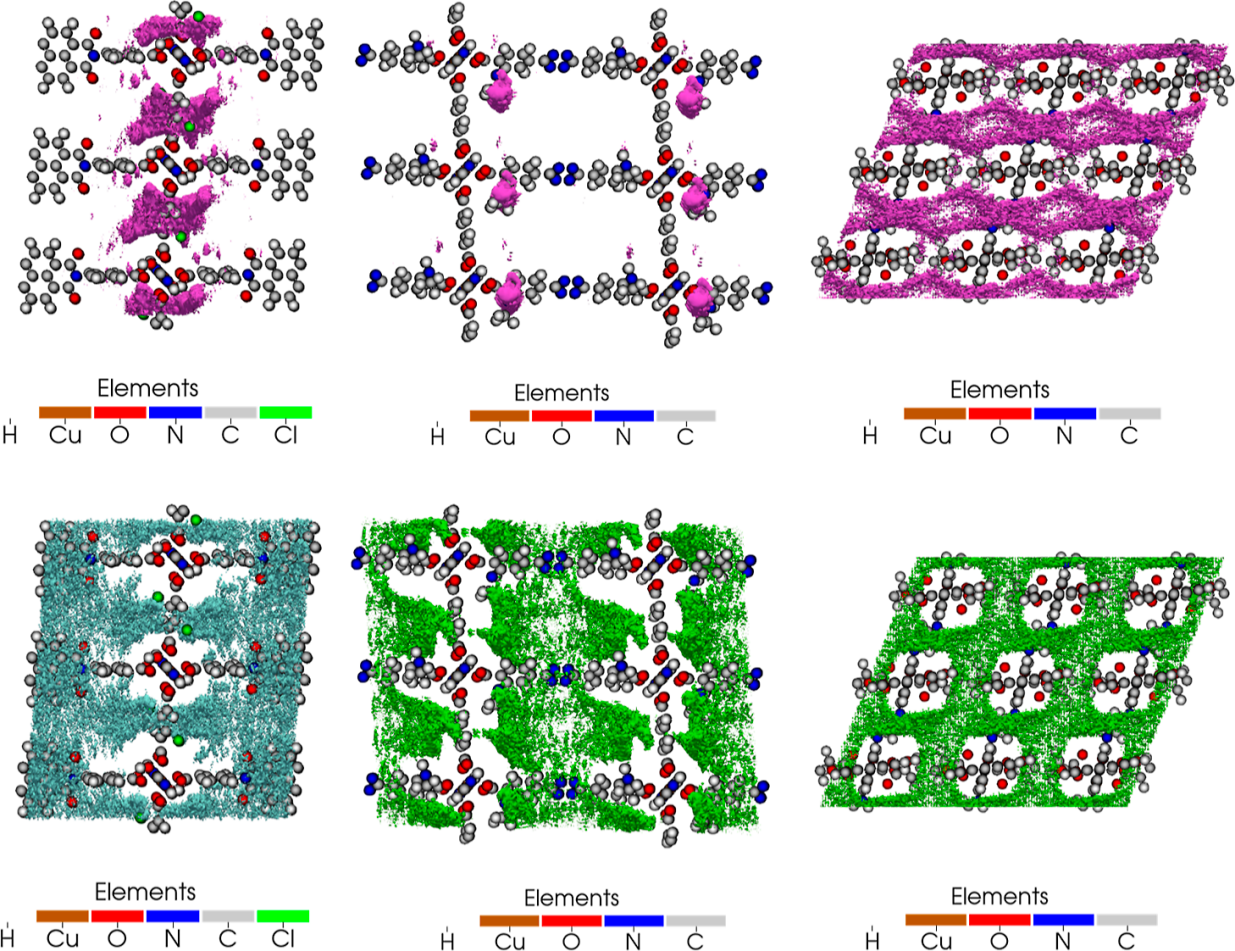
SO_2_ (top) and CH_4_ (bottom) density profiles in m2_o12_o29_pcu.260 (left), m2_o12_o29_pcu.221 (middle), and m2_o12_o27_pcu.188 (right) (at 0.1 bar, 298 K) (purple and green/cyan regions represent SO_2_ and CH_4_ occupation regions).

Figure 5 shows the adsorbate density profiles obtained from GCMC simulations at 0.1 bar, 298 K in the top three materials for SO_2_/CH_4_ separation in which SO_2_ molecules are relatively more localized near the pores, whereas CH_4_ molecules are typically dispersed throughout the material. Examining the SO_2_ and CO_2_ density profiles in the top three materials in Figure S6, it can be inferred that SO_2_ molecules prefer the pore corners while the distribution of weaker adsorbing sorbate, CO_2_, can be narrower (compared to CH_4_ in the SO_2_/CH_4_ mixture). For the SO_2_/N_2_ separation, the density profiles in Figure S7 are akin to those for the SO_2_/CH_4_ separation where the relatively small quadrupole moment of N_2_ does not lead to localized N_2_ regions. The formation of SO_2_ clusters, which was attributed to the strong host–guest and dipole–dipole interactions between SO_2_ molecules,^15,16,26,72^ near the pore walls in all three gas separations are in line with the observations made for IRMOF-10, MFM-300(Al), MFM-601, M(bdc)(ted)_0.5_ (M = Ni, Zn), and SIFSIX materials.^15,26,72−74^ As RDFs obtained at 0.1 bar, 298 K demonstrate (Figures S8–S10) that SO_2_ molecules are typically located close to the O atoms of the framework where S_SO_2__···O_host_ interactions stabilize the sorbates as the interactions between SO_2_ and O of furan linker in MIL-160 do.^24^ Since the SO_2_ molecules are about 3–4 Å away from H atoms of the framework, they can interact through hydrogen bonds as in NOTT-300, MFM-300(In), and SIFSIX materials, where interactions between O of sorbate and H of the framework contribute to the sorption.^26,28,75^ Similarly, in some of the top MOFs involving N or Cl atoms, SO_2_ molecules are at relatively close distances, which enables strong electrostatic interaction between S atom of the sorbate and N or Cl atoms of the framework. A similar observation has been made for MOF-808-His where SO_2_ interacts favorably with N atoms of the framework.^25^ While strong adsorption has been seen in the top materials, as reported earlier, some MOFs may be deprived of strong SO_2_ interaction sites such as MOF-808 whose gas uptake is limited.^25^ One strategy to render such MOFs efficient for SO_2_ capture is functionalization, as exemplified by MOF-808-His, underlining the importance of investigating the functionalized MOFs whose bare forms may not show significant gas uptake or separation capability.^25^ Through functionalization, the pores may provide stronger host–guest interactions as they can be narrower (leading to stronger confinement effects) or grafted favorable interaction sites for sorbates.

Having discussed the separation performances of hypothetical MOFs of interest, now, we advance to the performance benchmarks of them with highly SO_2_ selective materials reported in the literature. Liu et al.^14^ performed GCMC simulations for the SO_2_/CO_2_ separation in MIL-160 and MFM-300(Al) where their selectivities are predicted to reach 220 and 53, respectively, under ambient conditions. Savage et al.^28^ estimated the SO_2_/CH_4_, SO_2_/CO_2_, and SO_2_/N_2_ selectivities of MFM-300(In) under ambient conditions as 425, 60, and 5000, successively. Cui et al.^26^ reported the SO_2_/CH_4_, SO_2_/CO_2_, and SO_2_/N_2_ selectivity of SIFSIX materials using IAST and showed that SIFSIX-1-Cu and SIFSIX-2-Cu-i can attain high SO_2_/CH_4_ (SO_2_/N_2_) and SO_2_/CO_2_ selectivities of 992–1241 (2510–3145) and 86–89 under ambient conditions. Compared to the literature values, it can be deduced that there are many hypothetical MOFs identified in this work, which can be much more SO_2_ selective. While a holistic comparison involving all three separation performance metrics would have been more helpful, the foregoing studies do not report all metrics of interest.

Considering the well-defined structures of MFM-300(In) and SIFSIX-1-Cu and good agreement between the experimental and simulated SO_2_ uptakes in MFM-300(In) and SIFSIX-1-Cu (see Table S1), we calculated their SO_2_ gas separation performances (at the conditions specified in the Computational Methods section) as well to demonstrate the relative performances of hypothetical MOFs with respect to the synthesized MOFs. The structures of MFM-300(In) and SIFSIX-1-Cu were obtained from the literature.^28,76^ MFM-300(In) and SIFSIX-1-Cu were reported to have high SO_2_/CH_4_ (425 and 1241.4), SO_2_/CO_2_ (60 and 70.7), and SO_2_/N_2_ (5000 and 3145.7) selectivities calculated using IAST for binary mixtures involving 50 and 10% SO_2_ under ambient conditions, respectively.^26,28^ For all three gas separations, MFM-300(In) demonstrates highly SO_2_ selective behavior (SO_2_/CH_4_, SO_2_/CO_2_, and SO_2_/N_2_ selectivities of 3128.5, 181.1, and 24,937.1, respectively); however, its SO_2_ working capacity (0.6, 0.4, and 0.6 mol/kg, respectively) and regenerability (7.5, 6.1, and 8.2%, respectively) are low. In contrast, SIFSIX-1-Cu has somewhat less selectivity (SO_2_/CH_4_, SO_2_/CO_2_, and SO_2_/N_2_ selectivities of 803.7, 46.8, and 4423.7, respectively) but possess much higher SO_2_ working capacities (8.8, 6.8, and 8.9 mol/kg, respectively) and regenerabilities (82.4, 73.7, and 82.5%, respectively). Comparing these with the separation performance metric ranges of hypothetical MOFs, it can be inferred that there are many hypothetical MOFs that can be superior to MFM-300(In) and SIFSIX-1-Cu.

The separation performances of MOFs were also assessed using Δ*Q*, correlated with productivity, and a considerably different ranking of materials (compared to that obtained using adsorption selectivity, working capacity, and regenerability) is observed (see Table S2). While this may suggest that the identified top materials may not be the best performers at the process level, the Δ*Q* metric is derived using multiple assumptions (e.g., plug flow and fixed bed initially free of adsorbates), which may not be necessarily true for all separation operations. As the process-level performances rely on multiple parameters (e.g., pellet size, pellet porosity, etc.) and adsorbent bed configurations, the identified MOFs can perform well at the process level provided that the process parameters are optimized.^77,78^

While hydrophobic MOFs do not necessarily meet the separation goals^79,80^ and investigating water affinities of the MOFs was not one of our main goals, we calculated *K*_H_ and −Δ*H* values for H_2_O for the top performing materials, as listed in Table 1, to find clues about their potential use under humid conditions. Table S3 demonstrates that *K*_H_ and −Δ*H* values for H_2_O of the top materials at 298 K are higher than those of ZIF-8, a reference hydrophobic MOF (*K*_H_ = 2.1 × 10^–6^ mol/kg/Pa and −Δ*H* = 12.4 kJ/mol).^80^ This implies that due to the comparatively large water affinity of the top materials, H_2_O may compete with SO_2_ and deteriorate the separation performances of these materials. Therefore, the materials identified for selective SO_2_ removal in this work should be regarded as promising materials for dry gas mixtures but not necessarily for humid gas mixtures. However, it is worthwhile to note that separation processes are typically conducted using multiple stages.^81−83^ This is because many materials, when used as single adsorbents, are not capable of performing simultaneous removal of all undesired species and/or boast higher working capacities for undesired species than those of multiple adsorbents.^81−83^ Use of multiple beds also facilitates the regeneration in a continuous operation.^83^ Thus, the high-performing MOFs identified in this work can still find use in the separation of gas mixtures involving SO_2_ as long as H_2_O content is removed at an earlier stage in a multi-stage process. Similar processes have been previously proposed for the flue gas separation.^84−87^ Also, despite not being investigated in this work, the structural stability upon gas exposure is crucial for sustainable gas separation applications. These stability tests are typically carried out under dry conditions.^60,61^ In some cases while dry SO_2_ exposure does not degrade materials, humid SO_2_ exposure can cause the degradation, as shown for ZIF-8 and MIL-125, which underscores the importance of H_2_O removal from SO_2_ involving gas mixtures.^60,61^

To sum up, by breaking down the separation performance metric values into the functional groups of the hypothetical MOFs, it has been shown that the top materials for all three separations involve not only functionalized MOFs but also bare MOFs as the latter can excel at working capacity and/or regenerability, albeit not being the most selective group of MOFs. It is worthwhile to note that these gas mixtures may also have H_2_O content;^14^ however, the effect of H_2_O on the separation performances of MOFs is not investigated in this study due to the high computational cost of ternary mixtures involving H_2_O. Another aspect that can emerge during SO_2_ adsorption is the instability/phase transition of the MOF that may occur due to the potent interaction between SO_2_ and the MOF, which are beyond the scope of this work.^3,24,60,61,72,88−90^ As our study has shown that MOFs can be highly SO_2_ selective, regenerable, and possess large SO_2_ working capacities, these results will foster more research on the synthesis/generation/use of MOFs for the efficient SO_2_ capture from various mixtures.

## 4 Conclusions

This study focuses on the computational screening of a sheer number of hypothetical MOFs for the identification of promising candidates for SO_2_/CH_4_, SO_2_/CO_2_, and SO_2_/N_2_ separations using a multi-level approach. Starting with structure filtering based on geometrical properties, a list of bare MOFs is obtained to be employed in the first level of binary GCMC simulations from which the top bare MOFs are identified. In the second level, these bare MOFs and their functionalized variants are screened to determine the materials with the best overall separation performances for the separations of interest. This screening strategy have revealed potentially high-performing hypothetical MOFs for the separation of SO_2_/CH_4_, SO_2_/CO_2_, and SO_2_/N_2_ mixtures in terms of adsorption selectivity, working capacity, and/or regenerability. It is worthwhile to note that the top materials identified are those showing high (but not the highest) performances in terms of each separation performance metric implying a balanced selection of materials. Such selection criteria can help reduce the risk of shortlisting materials with unrealistically high selectivities that may arise due to the inaccuracy of the charge partitioning methods. While the entire data set screened is composed of hypothetical MOFs, we anticipate that the impressive separation performances of the top materials would trigger further experimental and theoretical research on them regarding their synthesis, characterization, and testing.

## Supporting Information Available

Comparison of experimental and simulated adsorption data; structure–property relationships for SO_2_/CH_4_, SO_2_/CO_2_, and SO_2_/N_2_ separation; rankings of top MOFs based on separation potential (Δ*Q*); water affinities of top MOFs; adsorbate density profiles for the top three MOFs for SO_2_/CO_2_ and SO_2_/N_2_ separation; and RDF plots for the top three MOFs for all three gas separations (PDF)
Binary GCMC simulation data for SO_2_/CH_4_, SO_2_/CO_2_, and SO_2_/N_2_ separation (Labels with _x (_y) represent quantities obtained at adsorption (desorption) conditions) (XLSX)
