# Supplementary material for: Multi-Level Computational Screening of in Silico Designed MOFs for Efficient SO2 Capture
Source: J Phys Chem C Nanomater Interfaces. 2022 Jun 3;126(23):9875–88. doi: 10.1021/acs.jpcc.2c00227 (PMC9207907; doi:10.1021/acs.jpcc.2c00227)
Supplement: Supplementary file 1 — jp2c00227_si_001.pdf [file jp2c00227_si_001.pdf]

Supporting Information

Multi-Level Computational Screening of In Silico Designed MOFs for Efficient SO_2 Capture

Hakan Demir*, and Seda Keskin*

Department of Chemical and Biological Engineering, Koc University, 34450 Istanbul, Turkey

Table of Contents

1. Comparison of Experimental and Simulated Adsorption Data 3

Table S1. Experimental and simulated SO_2 uptake in MFM-300(In) and SIFSIX-1-Cu at 298 K, 1 bar. 3

2. Structure-Property Relationships 3

Figure S1. SO_2/CH_4 separation performances of bare hypothetical MOFs in tandem with their structural properties. 3

Figure S2. SO_2/CO_2 separation performances of bare hypothetical MOFs with respect to their structural properties. 4

Figure S3. SO_2/CO_2 separation performances of top 50 bare MOFs and their functionalized counterparts as a function of their structural features. 5

Figure S4. SO_2/N_2 separation performances of bare hypothetical MOFs in tandem with their structural properties. 6

Figure S5. SO_2/N_2 separation performances of top 50 bare MOFs and their functionalized counterparts with respect to their textural features. 7

3. Rankings of Top MOFs based on Separation Potential (ΔQ) 8

Table S2. Rankings of top MOFs as determined using the summation of adsorption selectivity, working capacity, and regenerability rankings and separation potential (ΔQ). 8

4. Water Affinities of Top MOFs 9

Table S3. H_2O Henry's constants (K_H) and enthalpy of adsorption at infinite dilution (-ΔH) at 298 K. 9

5. Adsorbate Density Profiles 10

Figure S6. SO_2 (top) and CO_2 (bottom) density profiles in m2_o11_o17_pcu.118 (left), m2_o11_o17_pcu.143 (middle), and m2_o11_o17_pcu.95 (right) (at 0.1 bar, 298 K). (Purple and green regions represent SO_2 and CO_2 occupation regions.) 10

Figure S7. SO_2 (top) and N_2 (bottom) density profiles in m2_o12_o18_pcu.79 (left), m2_o12_o29_pcu.249 (middle), and m2_o12_o29_pcu.260 (right) (at 0.1 bar, 298 K). (Purple and green/cyan regions represent SO_2 and N_2 occupation regions.) 11

6. Radial Distribution Function (RDF) Plots 12

*Corresponding authors: Hakan Demir: hakdemir@ku.edu.tr, Seda Keskin: skeskin@ku.edu.tr

Figure S8. Normalized RDF plots of the sorbates for the SO_2/CH_4 mixture at 0.1 bar, 298 K in m2_o12_o29_pcu.260 (top), m2_o12_o29_pcu.221 (middle), and m2_o12_o27_pcu.188 (bottom). 12
Figure S9. Normalized RDF plots of the sorbates for the SO_2/CO_2 mixture at 0.1 bar, 298 K in m2_o11_o17_pcu.118 (top), m2_o11_o17_pcu.143 (middle), and m2_o11_o17_pcu.95 (bottom). 13
Figure S10. Normalized RDF plots of the sorbates for the SO_2/N_2 mixture at 0.1 bar, 298 K in m2_o12_o18_pcu.79 (top), m2_o12_o29_pcu.249 (middle), and m2_o12_o29_pcu.260 (bottom). 14

1. Comparison of Experimental and Simulated Adsorption Data

Table S1. Experimental and simulated SO_2 uptake in MFM-300(In) and SIFSIX-1-Cu at 298 K, 1 bar.

Structure	Experimental Loading (mol/kg)	Simulated Loading (mol/kg)
MFM-300(In)	8.28	7.79
SIFSIX-1-Cu	11.01	11.85

2. Structure-Property Relationships

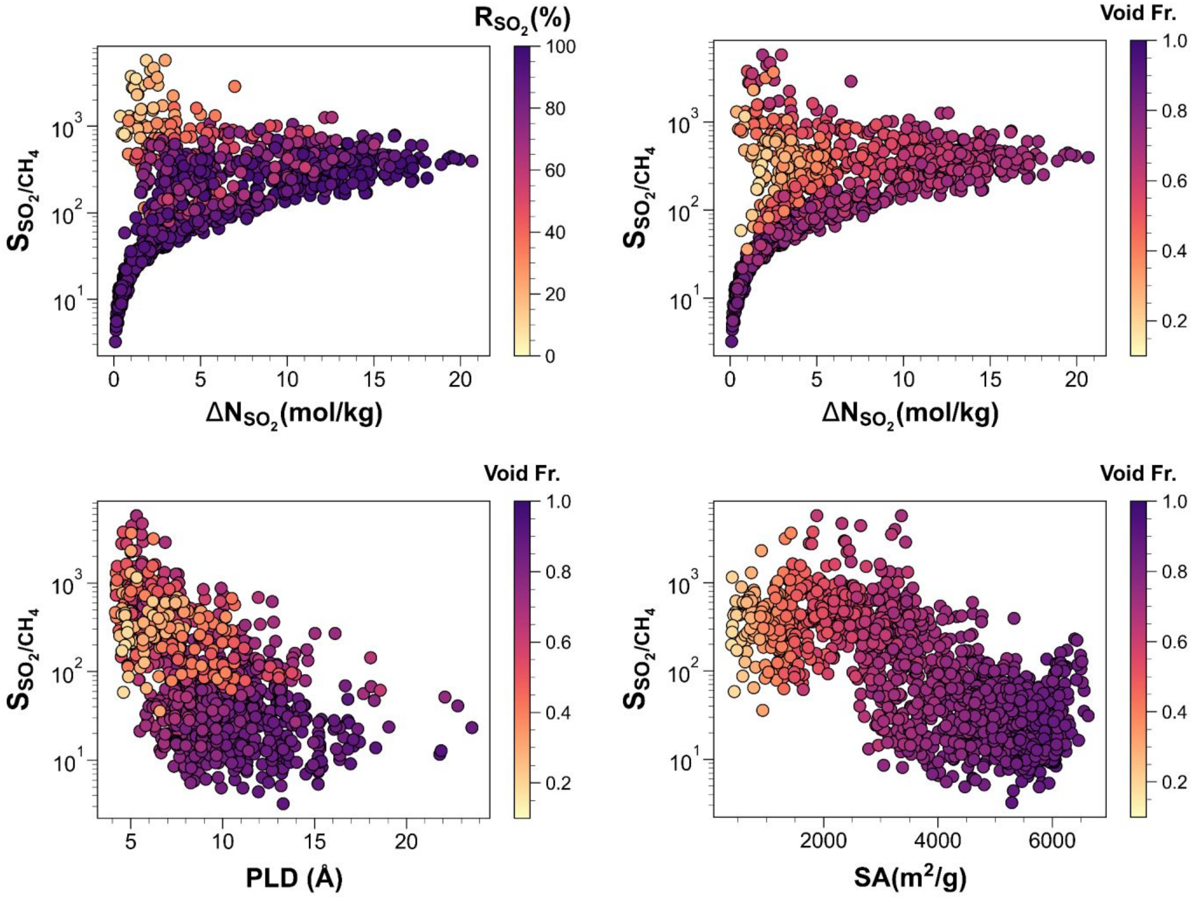

Figure S1. SO_2/CH_4 separation performances of bare hypothetical MOFs in tandem with their structural properties.

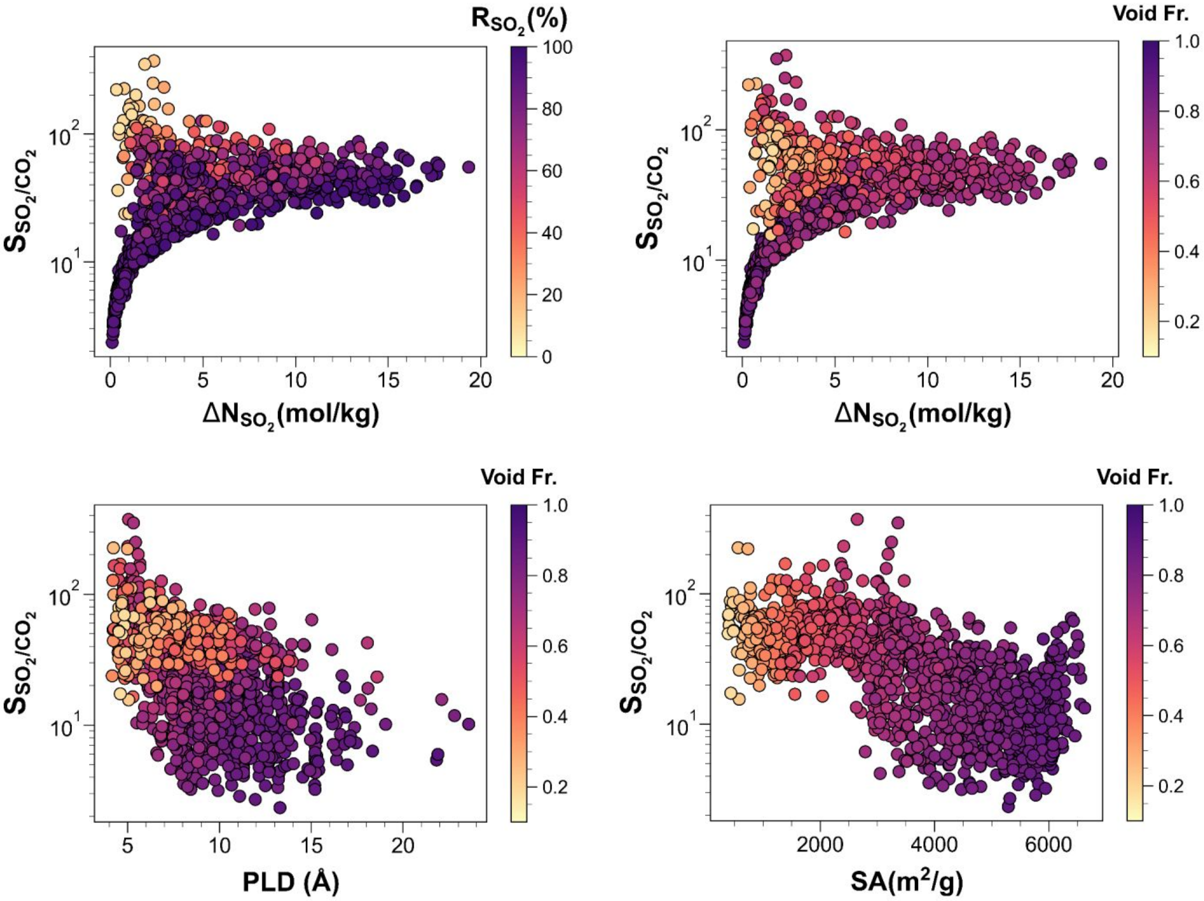

Figure S2. SO_2/CO_2 separation performances of bare hypothetical MOFs with respect to their structural properties.

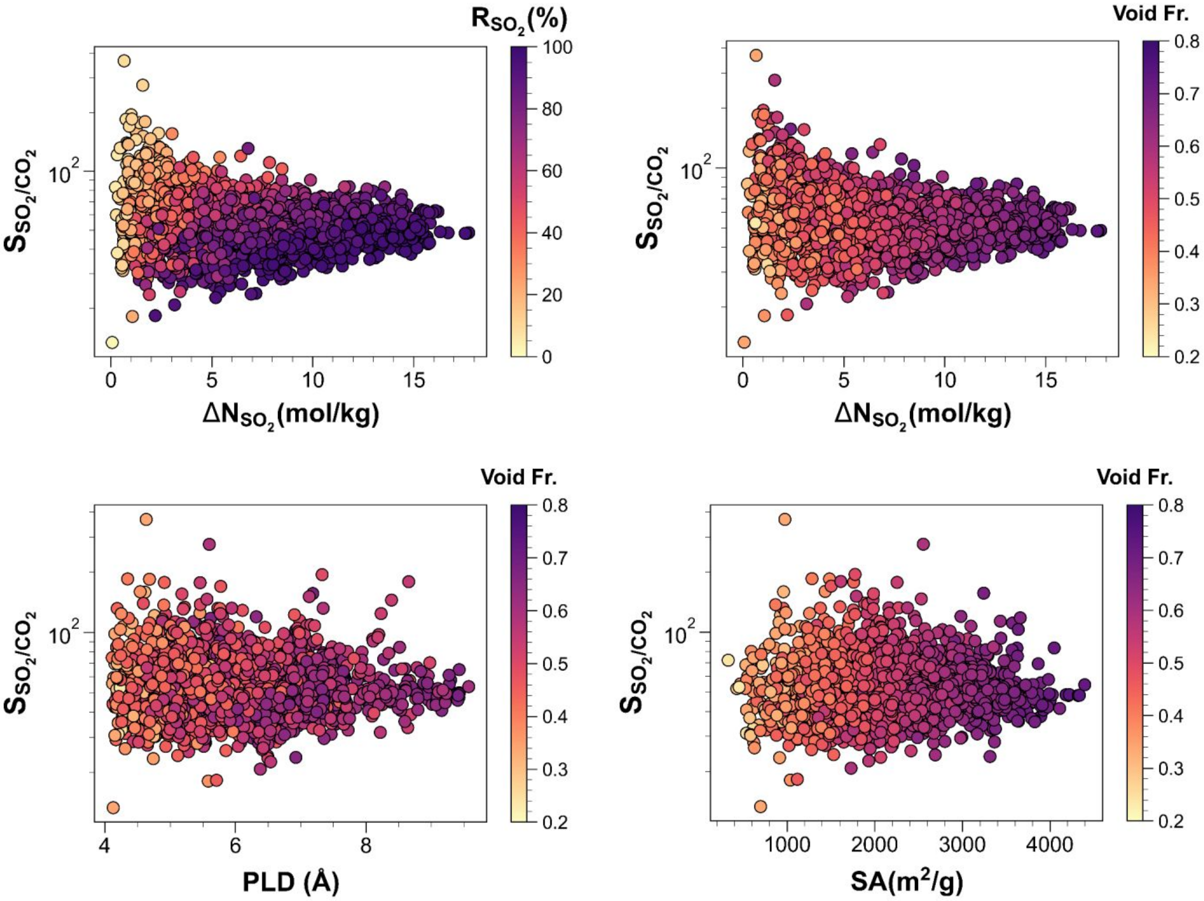

Figure S3. SO_2/CO_2 separation performances of top 50 bare MOFs and their functionalized counterparts as a function of their structural features.

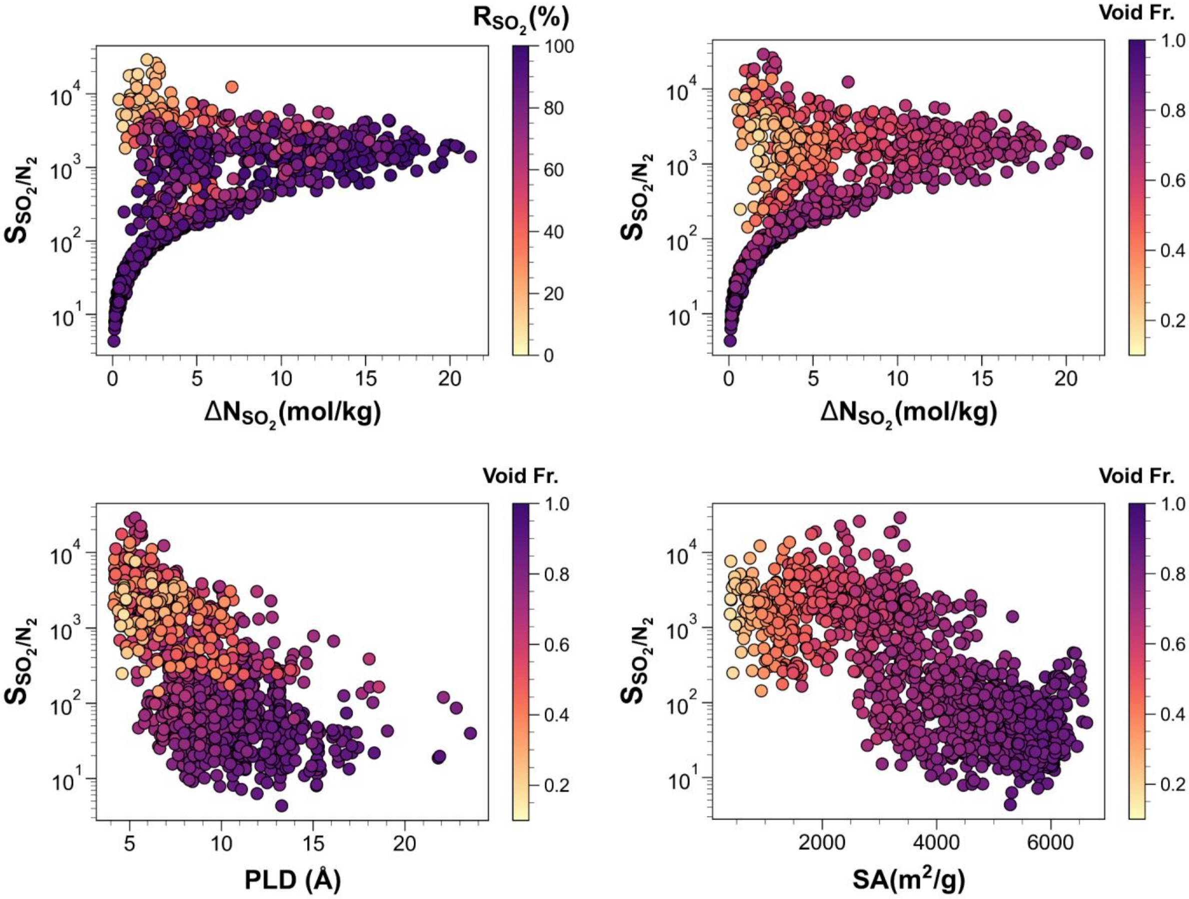

Figure S4. SO_2/N_2 separation performances of bare hypothetical MOFs in tandem with their structural properties.

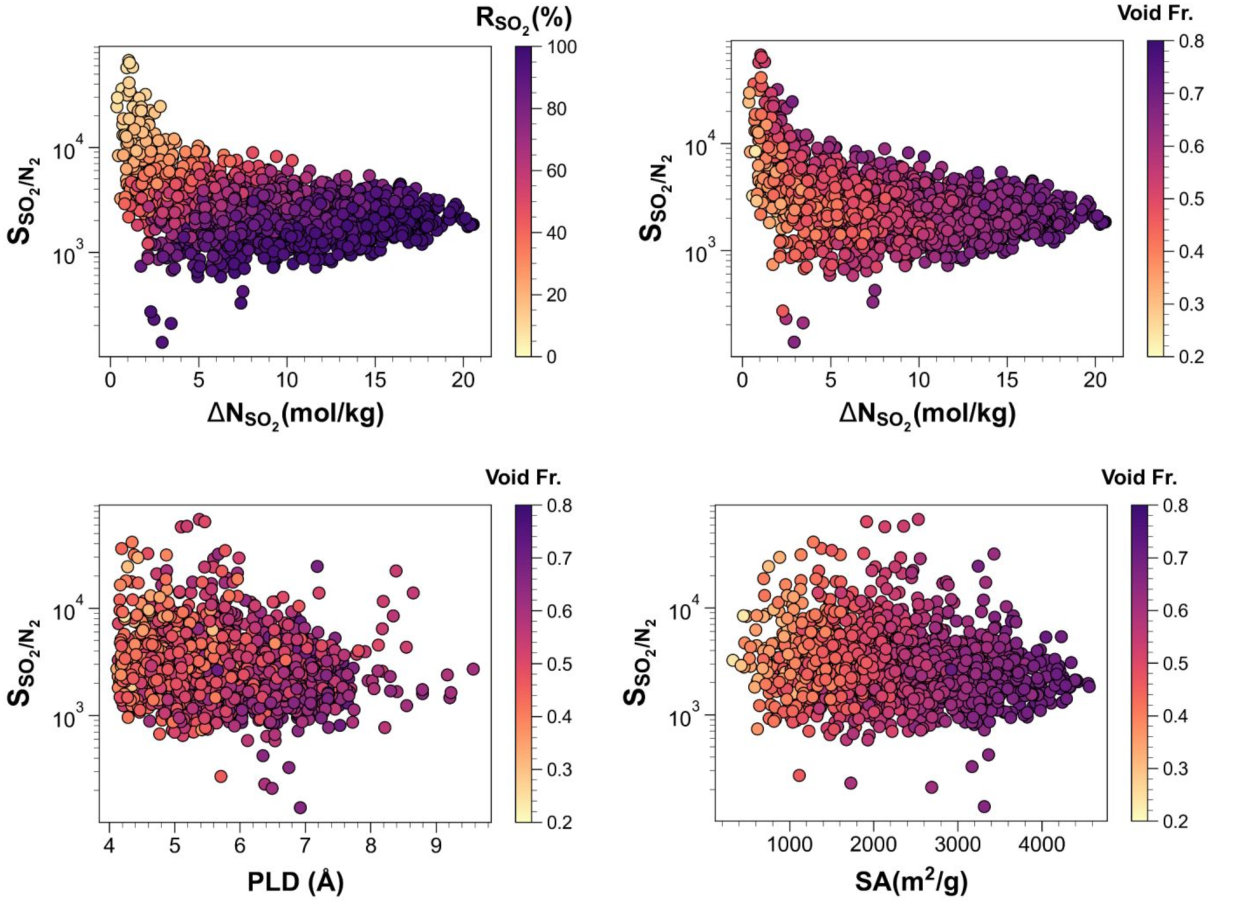

Figure S5. SO_2/N_2 separation performances of top 50 bare MOFs and their functionalized counterparts with respect to their textural features.

3. Rankings of Top MOFs based on Separation Potential (ΔQ)

Table S2. Rankings of top MOFs as determined using the summation of adsorption selectivity, working capacity, and regenerability rankings and separation potential (ΔQ).

Structure	Overall Rankings	ΔQ-based Rankings
	SO_2/CH_4	
m2_o12_o29_pcu.260	1	23
m2_o12_o29_pcu.221	2	25
m2_o12_o27_pcu.188	3	108
m2_o12_o29_pcu.2	4	83
m2_o12_o27_pcu.168	5	117
m2_o12_o29_pcu.249	6	69
m2_o12_o29_pcu.85	7	84
m2_o12_o29_pcu.155	8	64
m2_o12_o29_pcu.157	9	50
m2_o12_o18_pcu.79	10	85
	SO_2/CO_2	
m2_o11_o17_pcu.118	1	65
m2_o11_o17_pcu.143	2	96
m2_o11_o17_pcu.95	3	44
m2_o17_o22_pcu.190	4	47
m2_o17_o22_pcu.29	5	46
m3_o12_o17_pcu.56	6	35
m2_o11_o17_pcu.246	7	73
m2_o11_o17_pcu.106	8	66
m2_o17_o22_pcu.257	9	39
m2_o11_o17_pcu.265	10	97
	SO_2/N_2	
m2_o12_o18_pcu.79	1	83
m2_o12_o29_pcu.249	2	52
m2_o12_o29_pcu.260	3	16
m2_o12_o18_pcu.84	4	25
m2_o12_o18_pcu.195	5	18
m2_o12_o18_pcu.162	6	126
m2_o12_o18_pcu.87	7	27
m2_o12_o18_pcu.46	8	29
m2_o12_o18_pcu.4	9	26
m2_o12_o18_pcu.42	10	22

4. Water Affinities of Top MOFs

Table S3. H_2O Henry's constants (K_H) and enthalpy of adsorption at infinite dilution (-ΔH) at 298 K.

Structure	K_H (mol/kg/Pa)	-ΔH (kJ/mol)
**Top 10 MOFs for SO_2/CH_4 separation**		
m2_o12_o29_pcu.260	1.20×10^{-3}	39.1
m2_o12_o29_pcu.221	3.30×10^{-4}	33.8
m2_o12_o27_pcu.188	5.90×10^{-4}	33.7
m2_o12_o29_pcu.2	2.80×10^{-4}	34.4
m2_o12_o27_pcu.168	3.20×10^{-4}	33.8
m2_o12_o29_pcu.249	1.34×10^{-3}	45.8
m2_o12_o29_pcu.85	1.15×10^{-4}	30.4
m2_o12_o29_pcu.155	1.22×10^{-4}	30.5
m2_o12_o29_pcu.157	7.44×10^{-4}	37.9
m2_o12_o18_pcu.79	1.45×10^{-4}	34.1
**Top 10 MOFs for SO_2/CO_2 separation**		
m2_o11_o17_pcu.118	2.24×10^{-4}	33.2
m2_o11_o17_pcu.143	1.62×10^{-4}	32.2
m2_o11_o17_pcu.95	4.61×10^{-4}	35.4
m2_o17_o22_pcu.190	5.25×10^{-4}	40.4
m2_o17_o22_pcu.29	8.21×10^{-4}	43.3
m3_o12_o17_pcu.56	1.63×10^{-4}	32.6
m2_o11_o17_pcu.246	2.09×10^{-4}	31.8
m2_o11_o17_pcu.106	5.75×10^{-4}	37.4
m2_o17_o22_pcu.257	8.35×10^{-4}	43.4
m2_o11_o17_pcu.265	4.61×10^{-4}	38.0
**Top 10 MOFs for SO_2/N_2 separation**		
m2_o12_o18_pcu.79	1.45×10^{-4}	34.1
m2_o12_o29_pcu.249	1.34×10^{-3}	45.8
m2_o12_o29_pcu.260	1.20×10^{-3}	39.1
m2_o12_o18_pcu.84	1.67×10^{-4}	32.2
m2_o12_o18_pcu.195	1.73×10^{-4}	32.7
m2_o12_o18_pcu.162	1.98×10^{-5}	24.0
m2_o12_o18_pcu.87	1.67×10^{-4}	32.2
m2_o12_o18_pcu.46	1.75×10^{-4}	32.7
m2_o12_o18_pcu.4	1.67×10^{-4}	32.2
m2_o12_o18_pcu.42	1.70×10^{-4}	32.2

5. Adsorbate Density Profiles

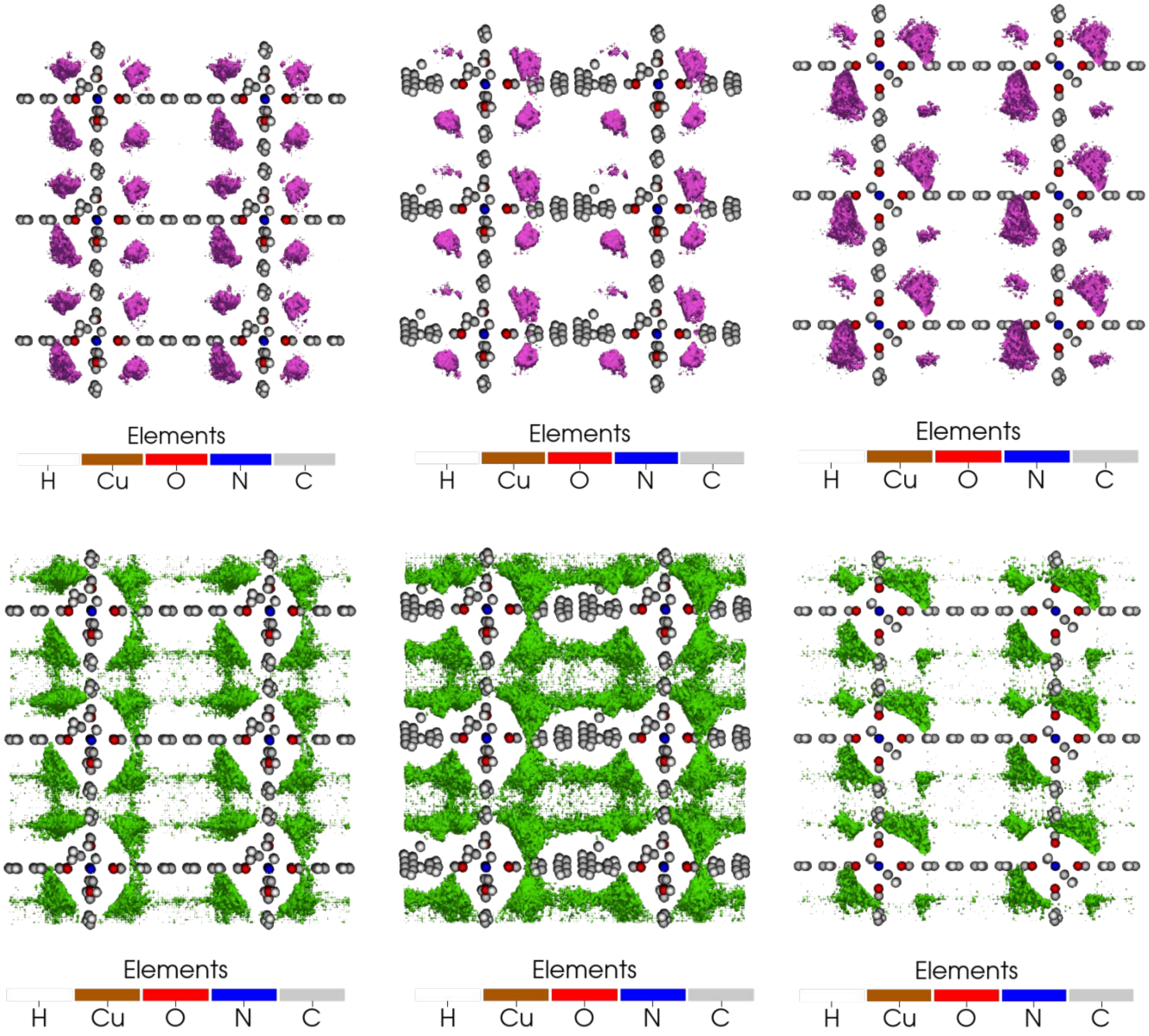

Figure S6. SO_2 (top) and CO_2 (bottom) density profiles in m2_o11_o17_pcu.118 (left), m2_o11_o17_pcu.143 (middle), and m2_o11_o17_pcu.95 (right) (at 0.1 bar, 298 K). (Purple and green regions represent SO_2 and CO_2 occupation regions.)

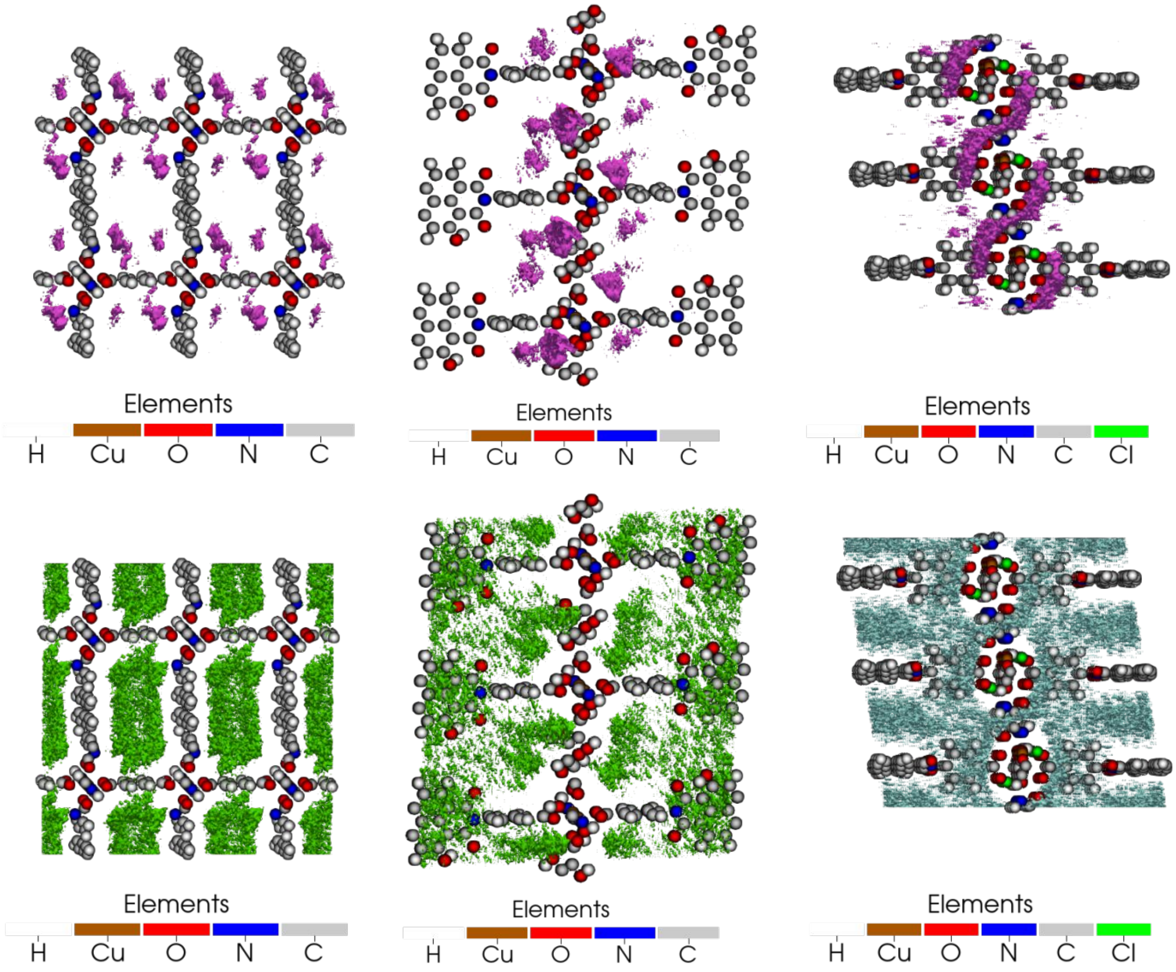

Figure S7. SO_2 (top) and N_2 (bottom) density profiles in m2_o12_o18_pcu.79 (left), m2_o12_o29_pcu.249 (middle), and m2_o12_o29_pcu.260 (right) (at 0.1 bar, 298 K). (Purple and green/cyan regions represent SO_2 and N_2 occupation regions.)

6. Radial Distribution Function (RDF) Plots

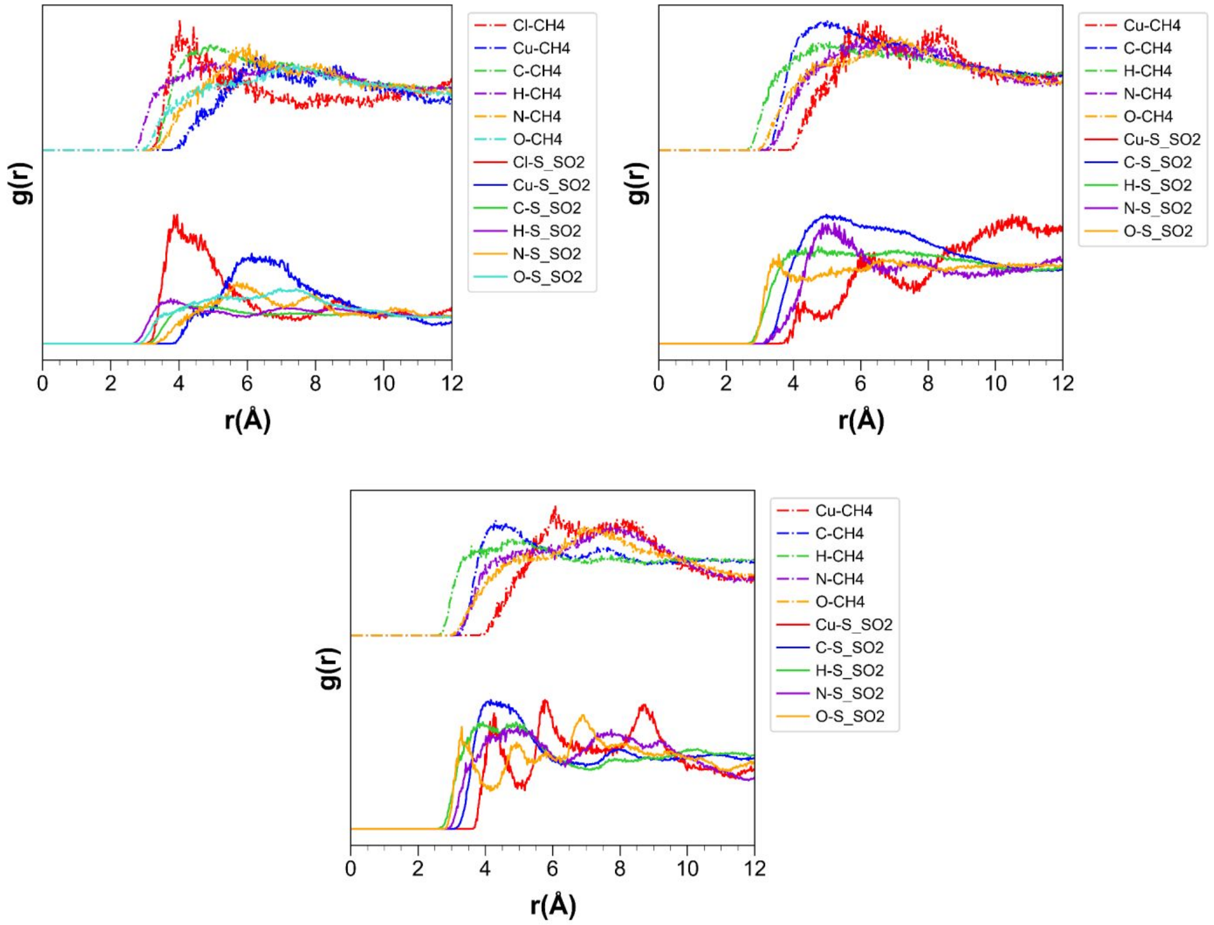

Figure S8. Normalized RDF plots of the sorbates for the SO_2/CH_4 mixture at 0.1 bar, 298 K in m2_o12_o29_pcu.260 (top), m2_o12_o29_pcu.221 (middle), and m2_o12_o27_pcu.188 (bottom).

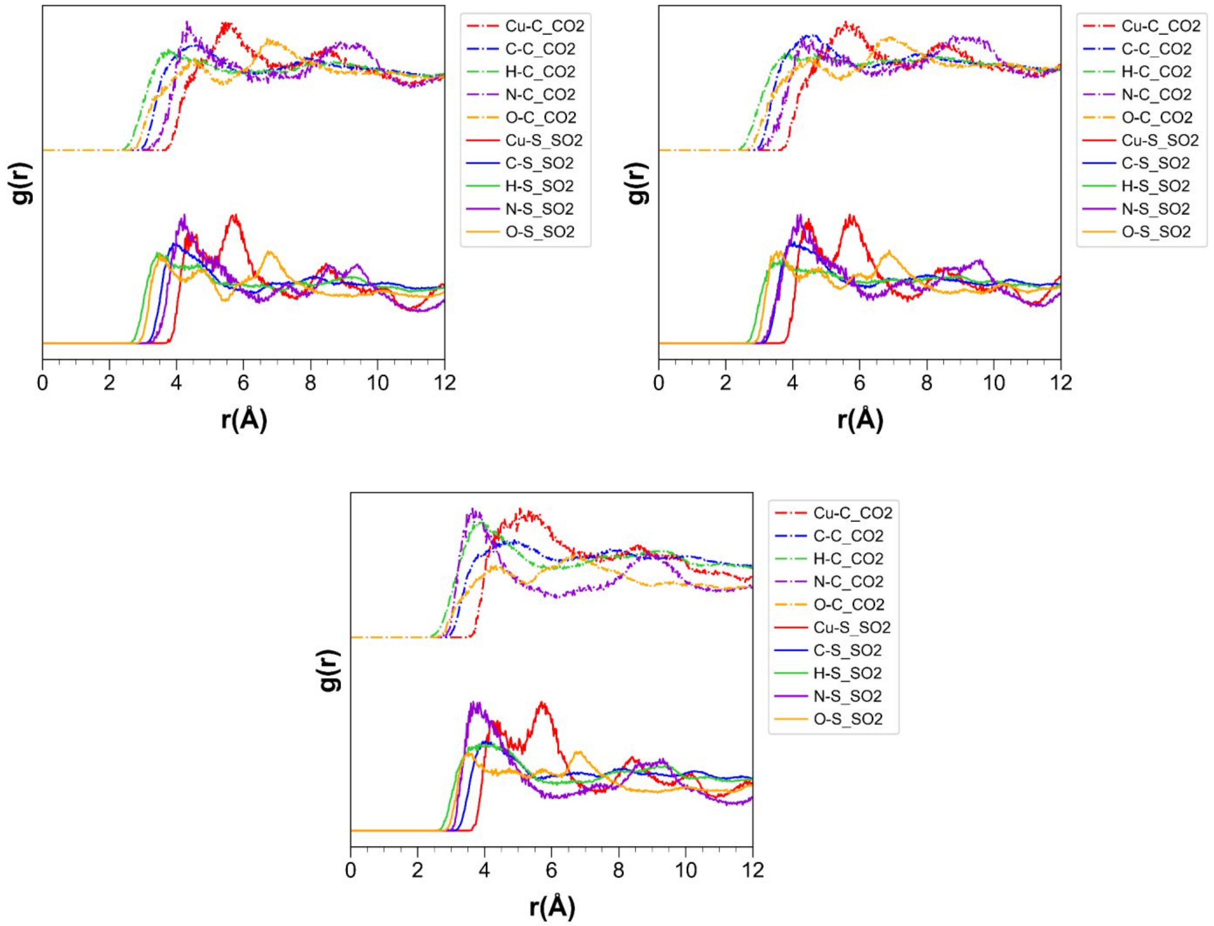

Figure S9. Normalized RDF plots of the sorbates for the SO_2/CO_2 mixture at 0.1 bar, 298 K in m2_o11_o17_pcu.118 (top), m2_o11_o17_pcu.143 (middle), and m2_o11_o17_pcu.95 (bottom).

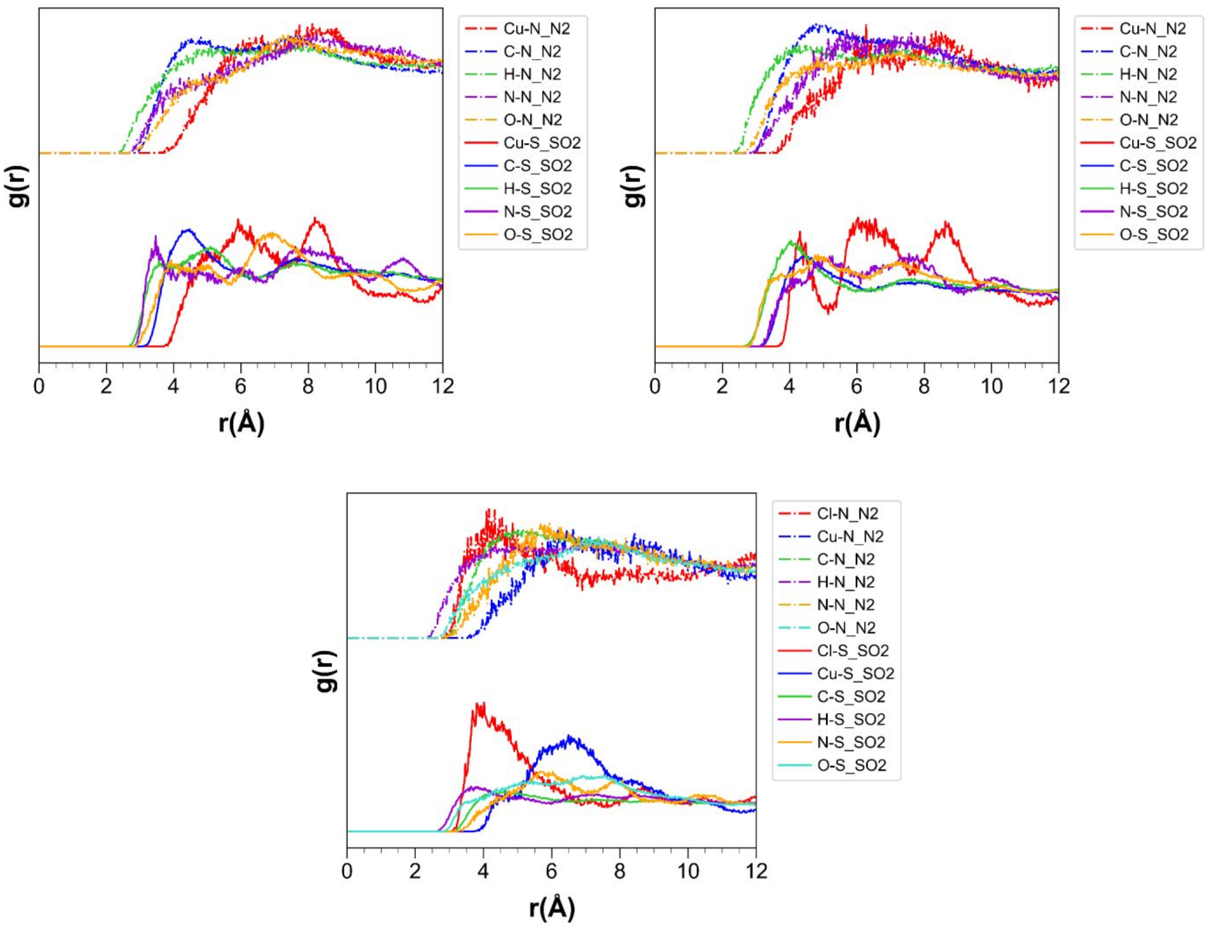

Figure S10. Normalized RDF plots of the sorbates for the SO_2/N_2 mixture at 0.1 bar, 298 K in m2_o12_o18_pcu.79 (top), m2_o12_o29_pcu.249 (middle), and m2_o12_o29_pcu.260 (bottom).
